# Discipline-specific open access publishing practices and barriers to change: an evidence-based review

**DOI:** 10.12688/f1000research.17328.2

**Published:** 2020-03-26

**Authors:** Anna Severin, Matthias Egger, Martin Paul Eve, Daniel Hürlimann

**Affiliations:** 1 Institute of Social and Preventive Medicine (ISPM), University of Bern, Bern, 3012, Switzerland; 2 Swiss National Science Foundation, Bern, 3001, Switzerland; 3 Department of English and Humanities, Birkbeck University of London, London, WC1H 0PD, UK; 4 Research Center for Information Law, University of St.Gallen, St.Gallen, 9000, Switzerland

**Keywords:** Open Access, Open Science, Publishing, Scholarly Communication, Science Policy, Communication Technologies, Scientometrics, Meta-Synthesis

## Abstract

**Background: **Many of the discussions surrounding Open Access (OA) revolve around how it affects publishing practices across different academic disciplines. It was a long-held view that it would be only a matter of time before all disciplines fully and relatively homogeneously implemented OA. Recent large-scale bibliometric studies show, however, that the uptake of OA differs substantially across disciplines. We aimed to answer two questions: First, how do different disciplines adopt and shape OA publishing practices? Second, what discipline-specific barriers to and potentials for OA can be identified?

**Methods: **In a first step, we identified and synthesized relevant bibliometric studies that assessed OA prevalence and publishing patterns across disciplines. In a second step, and adopting a social shaping of technology perspective, we studied evidence on the socio-technical forces that shape OA publishing practices. We examined a variety of data sources, including, but not limited to, publisher policies and guidelines, OA mandates and policies and author surveys.

**Results: **Over the last three decades, scholarly publishing has experienced a shift from “closed” access to OA as the proportion of scholarly literature that is openly accessible has increased continuously. Estimated OA levels for publication years after 2010 varied between 29.4% and 66%. The shift towards OA is uneven across disciplines in two respects: first, the growth of OA has been uneven across disciplines, which manifests itself in varying OA prevalence levels. Second, disciplines use different OA publishing channels to make research outputs OA.

**Conclusions: **We conclude that historically rooted publishing practices differ in terms of their compatibility with OA, which is the reason why OA can be assumed to be a natural continuation of publishing cultures in some disciplines, whereas in other disciplines, the implementation of OA faces major barriers and would require a change of research culture.

## Introduction

As a response to perceived limitations of the subscription-based model of scholarly publishing and propelled by technical possibilities provided by the internet and the world wide web, Open Access (OA) presents a new model of academic publishing ^1^. OA takes different forms but generally offers free and unrestricted access to the outputs of academic research with relaxed constraints on reuse, as opposed to publications being behind subscription paywalls and under copyright ^2^. Having gained global recognition, the potential implications of OA for academic publishing continue to generate debate in the academic community. Many of these discussions revolve around how OA affects publishing practices in different academic disciplines ^3^.

The foundation for OA was laid in high-energy physics when Paul Ginsparg established the arXiv open repository for preprints ^4^. OA soon appeared to constitute an “inescapable imperative” ^5^ for several reasons: first, OA gained early momentum based on a combination of grassroots advocacy initiatives promoting unrestricted access to publications and funders, universities and national governments implementing OA mandates and policies that require scholars to make their outputs publicly accessible ^6^. Second, OA has the potential to enhance scholarly communication by speeding up the dissemination of research outputs, by expanding readership and by increasing the impact of research outputs ^5, 7^. These trends suggested that it would only be a matter of time for all academic disciplines fully to adopt OA and to converge on a stable set of homogeneous OA publishing practices ^8^. In contrast to these expectations, recent bibliometric studies show that academic disciplines vary considerably in their OA publishing practices ^9, 10^.

Such bibliometric studies are in large part descriptive and, as such, do not analyse the mechanisms that shape discipline-specific OA publishing practices. This limitation becomes relevant as vast amounts of resources and efforts are committed to the development, maintenance and advancement of OA communication channels. In this article, we answer the following questions that pertain to this topic: (1) How do different academic disciplines adopt and shape OA publishing practices? (2) What discipline-specific barriers to and potentials for OA publishing can be identified? In order to answer these questions, we first synthesise relevant bibliometric studies that were aimed at assessing the prevalence and patterns of OA publishing practices across disciplines. Adopting a social shaping of technology perspective, we then develop an analytical framework that consists of socio-cultural and technological factors that generally shape publishing practices. We apply this analytical framework to the case of OA publishing and examine evidence on the forces that represent barriers to and potentials for OA. Doing so, we examine and aggregate evidence from a variety of primary data sources.

## Methods

### Definition of open access and open access routes

Fifteen years of research into the prevalence of OA have produced a number of different concepts of OA and its sub-types ^1^. One influential definition of OA is that offered by the 2002 Budapest Open Access Initiative, which understands scholarly outputs as OA if they are both free to read and free to reuse, without any financial, legal, or technical barriers other than gaining access to the internet ^11, 12^. However, a number of bibliometric studies have adopted a more lax definition of OA. Some require only that OA contents are freely available to read online, while disregarding reuse rights ^13– 16^. Others apply the minimum requirement that scholarly articles should be freely available to read online, and assess factors that determine their openness, for example what rights are provided by different types of licences or how articles are stored ^11, 17, 18^. Following the latter studies, this study understands OA as scholarly outputs that are free to read online, either on a journal website or through an open repository, and that might or might not be free to reuse. This definition assumes that OA is a spectrum that encompasses a range of components, which determine the degree of openness of a certain publication outlet ^19^. Different sub-types, so-called "routes" of OA, can be identified, depending on when and where scholarly articles are made available, who makes them available and what rights are provided by different types of licences ^17^. The following routes are included in this study’s definition of OA: Gold OA, Green OA, Hybrid OA, Delayed OA and Bronze OA. These routes differ in their openness and sustainability across fundamental aspects of OA – reader rights, reuse rights, copyrights, author posting rights and machine readability ^19^. Some of these routes enjoy general support as sub-types of OA while others remain controversial ^20^. Their definitions are given in Table 1. These routes are understood as exclusive categories and publisher-hosted content trumps self-archived content ^11^. This study does not include "Black OA", which refers to articles shared on illegal pirate sites, for example Sci-Hub, and "Academic Social Networks" (ASNs) or "Free availability" (FA), which describes authors sharing their papers on commercial online social networks like ResearchGate or other websites ^2^ ^11^. Where bibliometric studies differ from our definition of OA, this will be highlighted.

**Table 1.** Open access routes.

Open access route	Definition
Gold OA	Articles published in an OA journal, in which all articles are accessible directly and freely on the journal or publisher website, and which does charge publication fees ^11, 17^.
Platinum OA	Articles published in an OA journal, in which all articles are accessible directly and freely on the journal or publisher website, and which does not charge publication fees to the author since costs are met by one or more sponsoring organizations ^21^.
Green OA	Articles published in a subscription journal, but self-archived by other parties than the publisher, usually the authors themselves, in open repositories. Open repositories can be disciplinary or institutional repositories and articles may be either accepted versions or electronic preprints ^7, 11, 17^.
Hybrid OA	Articles published in a subscription journal but that are immediately free to read under an open license, in exchange for APCs paid by the author(s) ^11, 22^.
Delayed OA	Articles published in a subscription journal but that are free to read after an embargo period ^22^.
Bronze OA	Articles free to read on the journal or publisher website, but without a clearly identifiable license ^11, 19^.

### Prevalence and patterns of open access publishing practices: Meta-synthesis of bibliometric studies

**Figure 1.**
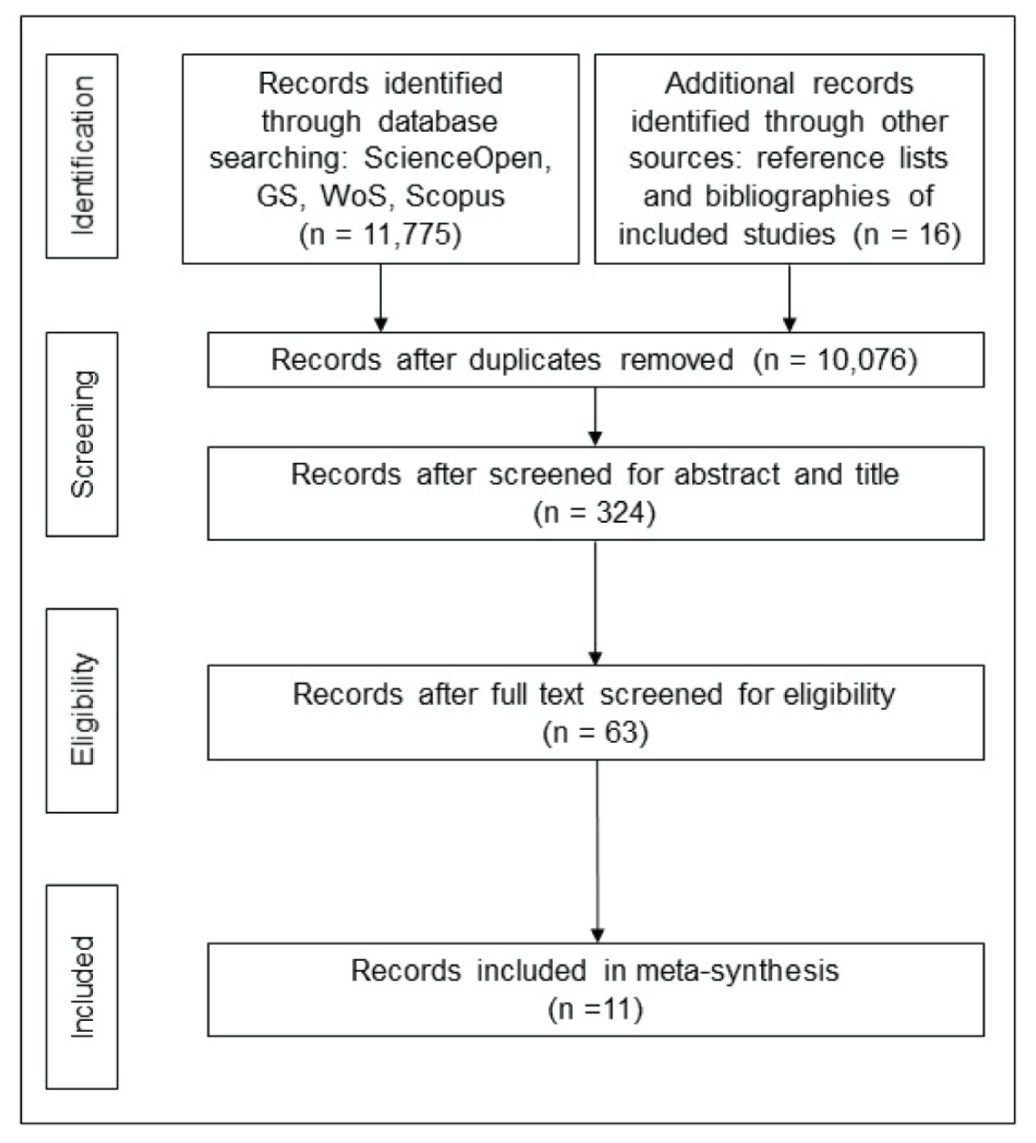
Search strategy.

The objective of our review is to identify and to synthesize bibliometric studies on the prevalence and patterns of OA publishing across academic disciplines. Such studies explore OA availability "bottom-up" through webbased queries of bibliometric databases such as Web of Science (WoS), Google Scholar (GS) or Scopus, and give uptake metrics for various OA routes. Because significant methodological differences can be identified within this approach, we conducted a meta-synthesis. The aim of a meta-synthesis is to integrate qualitatively, to compare, and to analyse methodologically heterogeneous studies, thereby allowing the emergence of interpretive themes ^23^. Here, we synthesised the results from bibliometric studies to identify patterns of OA publishing practices. The search was pre-planned and comprehensive, as it aimed to seek all available studies. No date limits were employed. The searches were conducted in August to October 2018 in a systematic way ( Figure 1). This involved, first, the querying of the online data bases ScienceOpen, Scopus, WoS and GS. The search was conducted using the following search string: “Discipline” AND “Publish*” AND “Open access” OR “OA”. The selection of the search terms was based on the topic literature. Second, reference lists and bibliographies of all included studies were evaluated for additional publications. Having identified key experts, their GS profiles were also searched for material. In an initial screening stage, two independent reviewers screened titles and abstracts of studies and decided on whether to include respective studies. Studies were excluded that did not meet our selection criteria ( Table 2). In a second screening stage, we assessed the full texts and extracted data on reported proportions of publications that were OA from the "Results" sections of included studies.

**Table 2.** Selection criteria literature search.

Criterion	Description
OA publishing practices	The study examines the overall prevalence of OA and the uptake of OA routes rather than only assessing the overall free availability of scholarly outputs.
Academic disciplines	The study examines OA publishing practices across broad academic disciplines, including the medical and life sciences, natural sciences, social sciences and law and humanities.
International scope	The study assesses OA publishing practices across countries. The scope is not limited to a national setting.
English language	The study is written in English.

### Mechanisms and factors shaping open access publishing practices: Narrative review of sociotechnological forces

Our goal in this section is to explain the patterns of OA publishing practices that we observed in the previous section. To do this, we performed a narrative review of the mechanisms and factors that shape OA publishing practices. We recruited an interdisciplinary team of researchers covering the natural and technical sciences, medicine, social sciences, law and the humanities. We did not perform a systematic review of the literature, but developed an analytical framework of socio-cultural and technical factors that shape publishing practices. Each co-author used this framework as a tool for identifying the socio-technical mechanisms and factors that shape OA publishing practices within their own discipline. The evidence included in this review was identified and selected through queries of online databases, including ScienceOpen, Scopus, WoS and GS. Reference lists and bibliographies of relevant studies were evaluated manually for additional evidence.

## Results

### Prevalence and patterns of open access publishing practices

The characteristics of the studies included in our review are presented in Table 3. In general, studies were concerned with the questions of (1) how much literature is OA across all disciplines and for individual disciplines, and (2) how much literature is published via different OA routes across all disciplines and for individual disciplines. Making use of automated web search strategies, studies assessed whether openly accessible versions of scholarly publications could be found on the web. Within this broad approach, important methodological differences can be identified. This relates to, first, definitions of OA and different OA routes. Some studies only estimated overall OA prevalence levels, but did not assess the relative uptake of different OA routes ^13– 15, 24, 25^. Others did assess the relative importance of Gold and Green OA, but not the uptake levels of other OA sub-types ^1, 9, 17^. One study assessed relative uptake levels for Gold, Green, Hybrid and Bronze OA, but excluded Delayed OA from its analyses ^11^. Two further studies estimated uptake levels for Delayed OA, but only as part of "Other OA", together with Hybrid OA, ASNs and other websites ^10^. A small number of studies included ASNs and FA in their definitions of OA, either as a part of Green OA together with other websites ^16, 17^, as the sub-type "Other OA" merged with Delayed OA, Hybrid OA and other websites ^10^, or as the sub-type "FA" together with other websites and harvesters ^18^. Second, bibliometric studies covered different publication years for which they determined OA prevalence levels, spanning 1992 ^24^ to 2017 ^13^. Third, in determining OA prevalence levels, studies used different databases and search strategies. Some studies examined the WoS database or its predecessor Thomson Reuters ISI Web of Knowledge in full ^13, 24, 25^, while others assessed random samples of papers indexed in these databases ^9, 11^, or combined them with Scopus ^17^ or the Social Sciences Citation Index and Humanities Citation Index ^18^. Another subset of studies examined Scopus either in full or as random samples of articles indexed therein ^1, 10, 14^, and one study used GS ^15^. In assessing whether openly accessible versions of scholarly publications indexed in these databases can be found, some studies searched for their corresponding freely available full text versions via Unpaywall ^13^, in the oaDOI database ^11, 25^ or in the 1science database of OA articles ^17^. Other studies searched for OA versions in GS ^14, 15^ or via Google, either manually ^1^ or by means of automated robot crawling ^9, 24^. Table 4 shows the main findings of the studies included in our meta-synthesis.

***Overall uptake on OA.*** The figures for the overall prevalence of OA show that OA levels have increased steadily across all disciplines, from 20.4% of all scholarly outputs reported as OA in 2008 (including ASNs and FA) ^16^, to 23% in 2010 ^9^ and more than one third of all scholarly outputs being OA in publication years later than 2010: 46.9% for publication years 2011 until 2013 (including ASNs and FA) ^10^, 54.6% on average in years 2009 and 2014 (including 20.7% ASNs and FA) ^18^, 36.1% on average between 2009 and 2015 ^11^, 66% for publication years between 2009 and 2017 ^13^, 54.8% in 2014 (including ASNs and FA) ^17^ and 29.4% in 2016 ^25^. We can distinguish between three phases. Dated between the late 1990s and the mid to late 2000s, the first phase can be characterised as a phase of formation: A few fields related to the natural and technical sciences took on a pioneering role in implementing OA, amongst these particularly mathematics, physics and space-related research fields, for which reported OA levels vary across studies between 23.5% for physics & astronomy (including ASNs and FA) and 42% for mathematics ^1, 9^. An exception to this are the fields engineering and chemistry, which feature consistently lower OA levels. The social sciences were also fast in embracing OA, featuring OA prevalence levels only slightly below those reported for the natural sciences ^1, 9, 24^. Medical fields were substantially slower in implementing OA than natural and social sciences ^1, 9, 24^. The second phase of OA is dated between the late 2000s and the mid 2010s and can be characterized as a period of transformation. For the early period of this phase, OA levels in the natural and technical sciences remained above those observed in other disciplines with reported OA levels between 27% for mathematics and 50% for computer sciences ^15^. In medicine, OA uptake soon increased substantially, causing OA levels in these fields to equal or surpass OA prevalence in other fields ^14^. Particularly biomedical research took on a leading role, featuring reported OA levels of 70.6% (including ASNs and FA) ^10^. During this period, the gap between the natural and technical sciences and medicine on the one side and the social sciences and humanities on the other side widened. The humanities and arts published research outputs to lesser degrees OA, featuring OA levels that vary across studies between 23.3% for visual and performing arts and 35.9% for general arts, humanities and social sciences (both including ASNs and FA) ^10^. The third phase of OA can be dated after the early 2010s and is a phase of stabilisation, in which differences in the OA publishing patterns across disciplines have become established. Studies consistently show that medical and health-related research fields are taking the leading roles in embracing OA, featuring reported OA uptake levels between 47.8% for clinical medicine and 85% for biomedical research ^11, 13, 17, 18, 25^. This is closely followed by physics, mathematics and earth and space sciences ^11, 13, 17, 18, 25^. OA uptake in the social sciences is close behind the natural sciences, followed by law, arts and humanities with some distance ^11, 13, 17, 18, 25^. Chemistry and engineering feature the lowest OA uptake levels, varying across studies between 15.5% and 35% for chemistry and between 17.4% and 29% for engineering ^11, 13^.

***Relative uptake of open access routes.*** Most OA is published via the Green route, featuring reported uptake levels that vary across studies between 5.9% (publication years 2011–2013), 21% (publication years 2005–2010) and 31% (publication year 2014, including ASN and FA) ^10, 17, 17^. Gold OA journals are also of importance for scholarly publishing, even though the relative uptake on Gold OA remains below Green OA for most publication years, with reported prevalence levels between 2% (publication years 2005–2010) and 12.1% (publication years 2011–2013) ^1, 10, 11, 14, 18^. Studies that also assessed the relative uptake on Bronze, Hybrid and Delayed OA have revealed that the importance of Bronze OA is comparable to that of Gold OA and that Hybrid and Delayed OA generally are of little importance for scholarly publishing, with less than 5% of all scholarly outputs being published Hybrid or Delayed OA ^11, 18^. ASNs and FA appear to play a highly relevant role for making research outputs openly accessible, featuring levels of 20.7% in 2009 and 2014 ^18^.

Looking at the relative importance of the different OA routes for each discipline, we observe that, for the medical sciences, publication in Gold and Bronze OA journals plays the most important role for making research findings OA, followed by Green OA and, with some distance, Hybrid and Delayed OA. For the natural and technical sciences, we see that there are substantial differences in the OA publishing patterns between different fields: scholars in physics, mathematics, astronomy and biology make large shares of their research outputs openly accessible through the Green route of OA, followed by Bronze OA, Gold OA, and, with some distance, Delayed and Hybrid OA. For scholars in chemistry and biology, Gold OA journals are of greater importance than any other OA route, followed by Green, Bronze and Hybrid OA. For scholars in the social sciences, Green OA is of greater importance for OA publishing than Gold OA, Bronze OA and Hybrid OA. In the humanities and law, scholars make research outputs openly accessible predominantly through publication of articles in Hybrid OA journals, followed by Green OA, Bronze OA and Gold OA ^1, 10, 11, 14, 17, 18^.

**Table 3.** Studies included in the meta-synthesis: Methodological approaches.

Study	Data sources	No. of analysed publications	Publication years	Definition of open access
Larivière and Sugimoto (2018)	Papers published between 2009 and 2017 that are indexed in WoS and have a DOI, combined with Unpaywall	12,683,296	2009 – 2017	Articles freely available to read, with two non-exclusive subcategories: Gold (available on a journal website) and Green (available in a repository). Bronze, Hybrid, Delayed and ASN/Other free availability not included.
Piwowar *et al*. (2018)	Random sample of recent journal articles indexed in WoS and with DOIs, combined with oaDOI database	100,000 articles	2009 – 2015	Publications free to read online, with four exclusive sub-categories: Gold (published in an OA journal that is indexed in the DOAJ); Green (toll-access on the publisher page, but with a free copy in an OA repository); Hybrid (free under an open license in a toll- access journal); Bronze (free to read on the publisher page, but without open license). ASN/Other free availability not included.
Bosman and Kramer (2018)	Full WoS database, combined with oaDOI database	12.3 million articles and reviews	2010 – 2017	Publications free to read online, with four exclusive sub-categories: Gold (published in an OA journal that is indexed in the DOAJ); Green (toll-access on the publisher page, but with a free copy in any OA repository); Hybrid (free under an open license in a toll-access journal) and Bronze (free to read on the publisher page, but without identifiable license). ASN/ Other free availability not included.
Science-Metrix (2018)	All articles in WoS and Scopus, combined with 1science database of OA articles	13.2 million articles	2006 – 2015	Articles available on the Internet in full-text form, that are freely readable and downloadable, with two non- exclusive sub-categories: Gold (made available for free by the publishers themselves, containing pure Gold, Bronze and Hybrid, or on the side of an aggregator) and Green (available in any repositories by parties other than publishers, includes Green only and ASN/Other free availability).
Martín-Martín *et* *al*. (2018)	All documents with a DOI from WoS, Social Sciences Citation Index and Arts & Humanities Citations Index, combined with GS	2.6 million documents	2009 and 2014	Freely online available publications, with four exclusive sub-categories: Gold (available in pure Gold journals listed in the DOAJ); Hybrid (available in journals not listed in the DOAJ, but with an OA license effective at the time of publication); Bronze (available in journals not listed in the DOAJ and without OA license); Green (available in institutional or subject repositories listed in ROAR and OpenDOAR); Delayed (available in journals with an embargo period) and ASN/Other free availability (available on websites, ASN, harvesters).
Jamali and Nabavi (2015)	First ten hits from queries of minor Scopus subject categories in GS	7,244 articles	1996 – 2013	Any free full-text version of articles accessible through GS, with two exclusive sub-categories (Gold and Green, not explicitly defined). No information on Bronze, Delayed, or Other free availability.
Khabsa and Giles (2014)	GS	Capture- recapture approach	No limit	Any free full-text version of articles accessible through GS, with two exclusive sub-categories (Gold and Green, not explicitly defined). No information on Bronze, Delayed, or ASN/Other free availability.
Archambault *et al*. (2014)	Scopus, combined with searches of DOAJ, ROAR, OpenDOAR, PubMedCentral, and other sources of freely downloadable papers	513,753 articles	1996 – 2013	Articles freely available to all, with three exclusive sub-categories: Gold (available in journals listed in the DOAJ, and on the PubMed Central list of journals); Green (available on institutional repositories as listed in OpenDOAR and/or in ROAR) and ASN/Other free availability (Delayed, Hybrid, available on authors’ web pages and elsewhere, in ASN and on aggregator sites).
Gargouri *et al*. (2012)	Random samples of articles indexed in Thomson- Reuters-ISI, combined with robot crawling web for OA full- texts	107,052 articles	1998 – 2006 and 2005 – 2010	Articles freely accessible online, with two exclusive sub-categories: Gold (articles freely accessible online in a journal) and Green (self-archived online and free for all copies of published work in any appropriate journal). No information on Bronze, Delayed, or ASN/Other free availability.
Björk *et al*. (2010)	Random sample of articles from Scopus, combined with Google searches for OA full-texts	1,837 articles	2008	Access to articles without any restrictions posed by subscriptions, with two exclusive sub-categories: Gold (articles published directly in OA journals) and Green (articles posted openly in any repositories or other web sites). ASN/Other free availability included as "other web sites" in Green. No information on Bronze or Delayed.
Hajjem (2006)	CDROM version of ISI’s Science and Social Science Citation Indices, combined with robot crawling of the web for OA full-texts	1,307,038 articles	1992 – 2003	Any full text accessible on the web, no sub-categories defined.

**Table 4.** Studies included in the meta-synthesis: Main findings.

Study	% OA by discipline (year)	% OA route by discipline (year)			
		Gold	Green	Hybrid	Bronze
Larivière and Sugimoto (2018)	All disciplines: 66% (2009 – 2017) Biomedical Research: 85% Clinical Medicine: 79% Health: 73% Mathematics: 67% Earth and Space: 57% Psychology: 56% Physics: 56% Biology: 51% Professional Services: 42% Social Sciences: 39% Chemistry: 35% Engineering and Technology: 29%	Not assessed	Not assessed	Not assessed	Not assessed
Piwowar *et al*. (2018) ^3^	All disciplines: 36.1% (2009 – 2015) Biomedical Research: 58.5% Mathematics: 52.7% Clinical Medicine: 47.8% Health: 41.8% Earth and Space: 40.4% Biology: 32.7% Physics: 31.6% Psychology: 29.7% Social Sciences: 25.1% Professional Fields: 20.6% Engineering and Technology: 17.4% Chemistry: 15.5%	All disciplines: 7.4% (2009 – 2015) Biomedical Research: 15.3% Health: 11.7% Mathematics: 11.2% Clinical Medicine: 10.3% Biology: 7.3% Earth and Space: 5.6% Psychology: 4.7% Engineering and Technology: 4.2% Physics: 3.1% Humanities: 3.0% Chemistry: 2.8% Arts: 2.4% Professional Fields: 1.4% Social Sciences: 1.3%	All disciplines: 11.5% (2009 – 2015) Physics: 23.6% Mathematics: 22.7% Social Sciences: 18.7% Psychology: 17.6% Health: 14.1% Professional Fields: 13% Biomedical Research: 10% Clinical Medicine: 9.8% Earth and Space: 8.5% Engineering and Technology: 8.3% Chemistry: 7.9 % Biology: 7.2% Humanities: 6.3% Arts: 4.9%	All disciplines: 4.3% (2009 – 2015) Mathematics: 9.4% Humanities: 8.6% Biomedical Research: 8.1% Clinical Medicine: 6.3% Biology: 4.2% Health: 3.0% Earth and Space: 2.7% Chemistry: 2.3% Physics: 2.1% Psychology: 2% Professional Fields: 1.8% Engineering and Technology: 1.8% Social Sciences: 1.8% Arts: 0.6%	All disciplines: 12.9% (2009 – 2015) Biomedical Research: 25.2% Earth and Space: 23.7% Clinical Medicine: 21.5% Biology: 14% Health: 13% Mathematics: 9.4% Arts: 6.7% Psychology: 5.4% Professional Fields: 4.4% Social Sciences: 3.3% Humanities: 3.2% Engineering and Technology: 3.2% Physics: 2.9% Chemistry: 2.5%
Bosman and Kramer (2018)	All disciplines: 29.4% (2016) Life Sciences & Biomedicine: 41.7% Social Sciences: 17.3% Physical Sciences/Technology: 14.8% Arts & Humanities: 13.9%	Not assessed	Not assessed	Not assessed	Not assessed
Science-Metrix (2018)	All disciplines: 54.8% (2014) Health Sciences: 59% Natural Sciences: 55% Applied Sciences: 47% Economic & Social Sciences: 44% Arts & Humanities: 24%	All disciplines: 23.3% (2014) Health Sciences: 33% Natural Sciences: 15% Applied Sciences: 13% Economic and Social Sciences: 8% Arts and Humanities: 7%	All disciplines: 31.5% (2014) Health Sciences: 33% Applied Sciences: 29% Natural Sciences: 15% Arts and Humanities: 9% Economic and Social Sciences: 8%	Not assessed	Not assessed
Martín-Martín *et al*. (2018) ^4^	All disciplines: 54.6% (2009, 2014) Medical and Life Sciences: 60% Natural Sciences: 50% Social and Behavioral Sciences: 49.9% Engineering Sciences: 40.2% Language, Information and Communication: 36.3% Law, Arts and Humanities: 32.3%	All disciplines: 7.3% (2009, 2014) Medical and Life Sciences: 8.2% Law, Arts and Humanities: 7.3% Language, Information and Communication: 5.7% Natural Sciences: 3.5% Engineering Sciences: 3.5% Social and Behavioral Sciences: 1.7%	All disciplines: 10.8% (2009, 2014) Medical and Life Sciences: 19.4% Social and Behavioral Sciences: 15.9% Natural Sciences: 15.3% Engineering Sciences: 8.7% Law, Arts and Humanities: 5% Language, Information and Communication: 4.4%	All disciplines: 1% (2009, 2014) Law, Arts and Humanities: 1.8% Medical and Life Sciences. 1.4% Language, Information and Communication: 1.2% Social and Behavioral Sciences: 0.5% Natural Sciences: 0.5% Engineering Sciences: 0.3%	All disciplines: 13.2% (2009, 2014) Medical and Life Sciences. 20.8% Natural Sciences: 7.5% Engineering Sciences: 3% Social and Behavioral Sciences: 5.3% Law, Arts and Humanities: 0.1% Language, Information and Communication: 0%
Jamali and Nabavi (2015)	All disciplines: 61.1% (2004 – 2014) Life Sciences: 66.9% Social Sciences: 60.8% Physical Sciences: 60% Health Sciences: 59.7%	Not assessed	Not assessed	Not assessed	Not assessed
Khabsa and Giles (2014)	All disciplines: 24% (all years) Computer Science: 50% Multidisciplinary Sciences: 43% Economics and Business: 42% Geosciences: 35% Physics: 35% Environmental Sciences: 29% Mathematics: 27% Medicine: 26% Biology: 25% Arts and Humanities: 24% Chemistry: 22% Social Sciences: 19% Agricultural Science: 12% Engineering: 12% Material Science: 12%	Not assessed	Not assessed	Not assessed	Not assessed
Archambault *et al.* (2014) ^5^	All disciplines: 46.9% (2011 – 2013) General Science & Technology: 89.7% Biomedical Research: 70.6% Mathematics & Statistics: 67.6% Biology: 66.2% Physics & Astronomy: 59.4% Earth & Environmental: 57.8% Psychology & Cognitive Sciences: 57.7% Public Health & Health Services: 57.2% Clinical Medicine: 56.3% Sciences Economics & Business: 54.9% Information & Communication Technology: 54.0% Agriculture, Fisheries & Forestry: 53.8% Social Sciences: 43.7% Enabling & Strategic Technologies: 39.3% Chemistry: 38.5% Built Environment & Design: 37.5% Arts, Humanities & Social Sciences: 35.9% Philosophy & Theology: 34.7% Engineering: 34.6% Historical Studies: 34.4% Communication & Textual Studies: 30.9% Visual & Performing Arts: 23.3%	All fields: 12.1% (2011 – 2013) Gen. Science & Technology: 58.0% Biology: 17.0% Agriculture, Fisheries & Forestry: 16.1% Public Health & Health Services: 15.8% Clinical Medicine: 14.8% Biomedical Research: 12.4% Information & Communication Technologies: 12.4% Mathematics & Statistics: 11.4% Chemistry: 9.5% Enabling & Strategic Technologies: 9.3% Social Sciences: 8.7% Communication & Textual Studies: 8.7% Earth & Environmental Sciences: 8.1% Historical Studies: 7.2% Psychology & Cognitive Sciences: 5.6% Economics & Business: 5.4% Philosophy & Theology: 5.1% Physics & Astronomy: 5.1% Engineering: 4.1% Built Environment & Design: 3.5% Visual & Performing Arts: 2.8% Gen. Arts, Humanities Social Sciences: 2.6%	All fields: 5.9% (2011 – 2013) Physics & Astronomy: 25.6% Mathematics & Statistics: 24.3% Economics & Business: 11.3% Information & Communication Technologies: 8.7% Earth & Environmental Sciences: 5.6% Social Sciences: 5.2% Philosophy & Theology: 5.1% Built Environment & Design: 4.6% Gen. Science & Technology: 3.9% Biology: 3.7% Psychology & Cognitive Sciences: 3.6% Communication & Textual Studies: 3.6% Gen. Arts, Humanities & Social Sciences: 3.5% Engineering: 3.2% Public Health & Health Services: 3% Visual & Performing Arts: 2.9% Agriculture, Fisheries & Forestry: 2.8% Enabling & Strategic Technologies: 2.7% Historical Studies: 2.5% Biomedical Research: 2.1% Clinical Medicine: 2.2% Chemistry: 1.8%	Not assessed	Not assessed
Gargouri *et al*. (2012)	All disciplines: 23% (2010) Mathematics: 42% Earth & Space: 37% Social Sciences: 37% Professional Fields: 29% Physics: 27% Engineering & Technology: 23% Psychology: 23% Biology: 22% Humanities: 19% Health: 17% Clinical Medicine: 14% Arts: 14% Biomedical Research: 12% Chemistry: 9%	All disciplines: 2% (2005 – 2010) Biomedical Research: 8% Clinical Medicine: 5% Health: 5% Mathematics: 2% Physics: 2% Earth & Space: 2% Biology: 2% Engineering & Technology: 1% Psychology: 1% Social Sciences: 1% Chemistry: 1% Arts: 1% Humanities: 1% Professional Fields: 1%	All disciplines: 21% (2005 – 2010) Mathematics: 43% Earth & Space: 36% Professional Fields: 29% Physics: 26% Engineering & Technology: 23% Social Sciences: 35% Psychology: 27% Biology: 22% Humanities: 14% Health: 12% Chemistry: 10% Clinical Medicine: 9% Arts: 9% Biomedical Research: 6%	Not assessed	Not assessed
Björk *et al*. (2012)	All disciplines: 20.4% (2008) Earth Sciences: 32.9% Mathematics: 25.6% Physics & Astronomy: 23.5% Social Sciences: 23.5% Medicine: 21.7% Biochemistry, Genetics & Molecular Biology: 19.9% Engineering: 18.4% Other areas related to Medicine: 15.2% Chemistry and Chemical Engineering: 12.9%	All disciplines: 8.5% (2008) Medicine: 13.9% Biochemistry, Genetics & Molecular Biology: 13.7% Other areas related to Medicine: 10.6% Mathematics: 8.1% Earth Sciences: 7% Social Sciences: 5.6% Chemistry and Chemical Engineering: 5.5% Engineering: 4.8% Physics & Astronomy: 3%	All disciplines: 11.9% (2008) Earth Sciences: 25.9% Physics & Astronomy: 20.5% Social Sciences: 17.9% Mathematics: 17.5% Engineering: 13.6% Medicine: 7.8% Chemistry and Chemical Engineering: 7.4% Biochemistry, Genetics & Molecular Biology: 6.2% Other areas related to Medicine: 4.6%	Not assessed	Not assessed
Hajjem *et al*. (2006)	All disciplines: Not reported (1992 – 2003) Sociology: 16% Biology: 15% Economics: 13.5% Business: 9% Management: 7% Psychology: 7% Health: 6.2% Political Science: 5.3% Education: 5.3% Law: 5.1%	Not assessed	Not assessed	Not assessed	Not assessed

### Analytical framework: Social shaping of technology

Previous studies have analysed discipline-specific publishing practices from a range of perspectives. In general, these perspectives originate from science and technology studies (STS), which look at how society, politics and culture shape research and technological innovation, and vice versa. Depending on their understanding of the direction of this relationship, these perspectives can be located on a scale that ranges from technological determinism at the one extreme to social constructionism of technology (SCOT) at the other extreme ^26^. Both positions have been shown to suffer from limitations in explaining scholarly publishing practices ^6^. The so-called “social shaping of technology” (SST) perspective that takes an intermediate standing between these extremes proves to be more useful for analysing publishing practices. SST is a theoretical stance that conceives the relationship between technology and society as one of mutual shaping ^27^. Technology is believed to be a social product patterned by the conditions of its creation and use ^28^. Central to technical change are choices made by social actors during the generation and implementation of new technologies ^29^. Which options social actors select is affected by both technical considerations and a range of social and cultural aspects. Thus, social choices influence the contents of technologies. At the same time, technologies have social implications as they shape human action and behaviour ^27^. Following this, scholarly publishing practices can be understood as socio-technical ensembles: the ways in which scholarly outputs are published is affected by the operational choices made by scholars during the implementation and use of communication technologies. These choices are influenced by both technical considerations and socio-cultural aspects. As communication technologies are implemented and used, they in turn affect the ways in which scholars communicate and disseminate their research findings. In order to explain discipline-specific OA publishing practices, it is necessary to examine the socio-cultural and technical factors that affect publishing choices. Based on these assumptions, we have developed an analytical framework that places focus upon technical factors and socio-cultural factors alike when analysing patterns of OA publishing practices ( Table 5).

**Table 5.** Framework of analysis.

Analytical Dimension	Description and Criteria
Author behaviour and attitudes	The publication outlets that scholars choose to publish the outputs of their research in and how they perceive these outlets, depending on the importance attached to the following criteria: a) quality control mechanisms and standards thereof b) speed of work and result-sharing c) impact of publication outlets d) prestige of publication outlets e) terms of academic promotion
Publisher behaviour and policies	The degrees to which publishers (i.e. commercial publishers, university presses, scholarly societies and others) decide to make full journal volumes or selected papers either closed access or OA and the timing of that, reflected in the following publishing policies and guidelines: a) lengths of embargoes b) policies on prior publication c) copyrights and licensing d) publishing costs
Infrastructures of scholarly communication channels	The characteristics of publication outlets (i.e. e-print servers and repositories, academic journals, digital libraries and academic social networks), described by: a) availability b) technical features c) uptake by scholars d) support by relevant stakeholders
Structural and institutional factors	Characteristics of research activities and conduct, described by: a) types of research (i.e. basic vs. applied research) b) types of work products and research outputs c) topic of research d) research costs e) funding structures f) copyrights and licensing
OA mandates and policies	The strength and effectiveness of OA mandates and policies by public funding agencies, research foundations and organisations, private companies and others, depending on their specific conditions: a) degree of obligation (i.e. mandate vs. recommendation) b) type of mandated or recommended OA route c) existence of “opt-out” opportunities for specific disciplines or research outputs d) permissible embargo periods for archiving in a repository following publication

### Open access in the medical sciences

Initially, medicine and health-related disciplines were reluctant to adopt OA publishing. From the mid-2000s onwards, the uptake on OA increased substantially and particularly biomedicine took on a leading role in embracing OA. Research outputs are predominantly made OA by publication in Gold OA journals. Hybrid OA, Bronze OA and Green OA are of less importance.

**A) Author behaviour and attitudes –** Several surveys and interview studies have shown that a large majority of authors support OA publishing, but the reputation of journals, impact factors, and quality and speed of peer review are more important factors determining the choice of publication outlets ^30– 33^. Surveys among academics from lower income countries indicate that the funding of APCs is an important concern, which might explain why authors from resource-limited settings are over-represented among publications in fraudulent journals that charge small fees but do not provide proper editorial and peer review services ^34– 36^.

**B) Publisher behaviour and policies –** As the OA model is unlikely to generate the same level of income and profit that can be achieved with the subscription model, commercial medical publishers have been reluctant to convert their subscription journals to OA ^3, 37^. The same applies to academic and professional societies ^38^. Some journals have now moved to allowing the self-archiving of submitted manuscripts without embargo periods, while for others self-archiving of accepted versions remains subject to embargo periods of 12 months. Pioneers among OA medical journals include the Journal of Clinical Investigation, which in 1996 became the first major journal to be freely available. Publication in the journal was free initially, but APCs were introduced after the journal lost 40% of its institutional subscribers ^39^. The BMJ followed in 1998, but moved some contents behind a paywall in 2005 ^40^. The number of OA journals increased considerably from 2000 onwards, with the rapid growth of OA publishers such as the not-for-profit publisher Public Library of Science (PLOS) or the commercial publisher BioMedCentral (BMC). The launch of OA journals by major biomedical research funders ^41– 43^ and the emergence of mega-journals are other factors that facilitate OA ^44^.

**C) Infrastructure of scholarly communication –** OA publishing focuses on Gold OA journals and only a small number of OA institutional and subject repositories has emerged. This is because, first, sufficient funding is available for publication in Gold OA journals. Second, journal publications are of central importance in academic hiring and promotion decisions. Third, there is a large number of high-quality Gold OA journals for authors to publish their research in. An exception to this is the PubMed Central (PMC), which archives full-text scholarly articles and has experienced rapid growth in the late 2000s to early 2010s as the National Institutes of Health (NIH) introduced an OA policy in 2008 that mandates its grantees to deposit the final peer-reviewed version of an article based on NIH-funded research in PMC. The embargo was initially 12 months after publication, but was later shortened to 6 months. Journals have since moved to be compliant with this Green OA mandate ^45^.

**D) Structural and institutional factors –** The main type of work products are journal articles. As research in the medical sciences and related fields mostly is funded by project-specific grants, it is fairly easy to integrate APCs into existing funding structures.

**E) Open access mandates and policies –** Evolving national and institutional OA policies and mandates have accelerated OA publishing. A substantial number of national governments have moved to require scholars to make their articles OA if based on publicly-funded research. Scholars can either follow the Gold or the Green route and are granted embargo periods of 6 or 12 months to comply with the latter ^13^. Major funders of (bio)medical research also play an active role in promoting OA. Since 2014, journal articles and book chapters based on research funded by the World Health Organization (WHO) have to be published in either an Gold or Hybrid OA journal or in a subscription journal that allows the depositing of accepted versions in PMC no later than 12 months after publication ^46^. The Wellcome Trust requires articles to be published in OA journals where a journal makes this option available and to be deposited as the accepted version in an open repositories no later than 6 months. Both funders provide repository infrastructures (PMC for NIH and PMC or PMC Europe for Wellcome Trust) and APC funds ^47^. The Wellcome Trust has launched its own OA journal, Wellcome Open Research, enabling its grant recipients to publish OA for free ^41^. In contrast to the USA, the policy environment in the UK favoured Gold and Hybrid OA, with particularly high uptake in the life sciences and increasing costs ^48^.

### Open access in the natural and technical sciences

For most publication years, the natural and technical sciences show the highest OA prevalence rates amongst all disciplines. There are differences in the publishing patterns between the sub-disciplines. Scholars in physics, mathematics, astronomy and information technology were early pioneers of OA. In biology, OA uptake increased in the early 2000s. Engineering and chemistry feature OA prevalence rates that are consistently lower.

**A) Author behaviour and attitudes –** The distribution of preprints has a long tradition in physics, mathematics, astronomy, and information technology. In biology, scholars initially were slower in embracing the idea of sharing preprints, but with the launch of platforms like PeerJ Preprints and bioRxiv in 2013, preprints took off ^49^. Surveys have revealed that, to scholars within these fields, rapid publication, high visibility and large readership appear to be the most important factors for choosing a publication outlet, and that scholars associate these features with repositories ^50, 51^. Adding to this, scholars generally show high levels of familiarity with OA ^16, 52^. In contrast, scholars in chemistry and engineering value publication in journals over self-archiving, causing Gold OA to play a bigger role than Green OA ^51^. Chemistry and engineering further show a particularly low uptake on OA. This might be because scholars have doubts about the quality of peer review in OA journals and are concerned that this might translate into low-quality publications ^53, 54^.

**B) Publisher behaviour and policies –** Commercial publishers and learned societies have been slow in embracing the idea of OA. This is because they face a potential loss of revenues in switching from a subscription model to an APC model ^55, 56^. Also, general concerns about the quality of OA journals are not only shared by scholars but also by publishers ^53^. As a result, most of the major commercial publishers as well as learned societies have been reluctant to convert their existing journals to OA or to set up new OA journals. An exception to this are few large publishing houses that set up new OA journals in disciplines that do not have a culture of preprint distribution, such as chemistry. In disciplines where there is a preprint culture, publishers are relaxing policies on prior publication and enable manuscripts deposited in repositories to be directly submitted to their journals ^53^.

**C) Infrastructure of scholarly communication –** In physics, mathematics, astronomy, information technology and, with some delay, in biology, scholars became used to sharing their research outputs openly making use of open repositories ^4^. Originally established within high energy physics, arXiv is the most popular repository and is now used by scholars in most fields of the natural sciences. Its concept has resulted in a number of discipline-specific repositories in other sub-fields, for example bioRXiv for biology ^49^. In the light of this publication culture, relatively few OA journals have emerged within these fields. In fields where there is a smaller culture of self-archiving in repositories, most particularly in chemistry and engineering, and initially in biology, the number of OA journals has grown slowly but steadily. These journals cover a variety of specific subject areas, are peer-reviewed, and, for the most part, published in English ^53, 57^. In biology, preprints finally took off after 2013 with the launch of platforms such as launch of PeerJ Preprints and bioRxiv ^49^.

**D) Structural and institutional factors –** The main types of work products are journal articles, preprints and conference proceedings. Researchers have reported that the process of self-archiving in repositories is easy and little time-consuming ^51^. Research is in large parts funded by project-specific grants, which would make it fairly easy for scholars to integrate APCs for Gold or Hybrid OA journals into existing funding structures. A structural factor that limits OA uptake particularly in chemistry and engineering, is that these fields are industry-oriented, which is incompatible with wide and open knowledge dissemination ^58^. This adds to the fact that, particularly within engineering, the focus is rather national than international as products are mostly produced for domestic markets ^58, 59^. Consequently, large numbers of publications are more practice-oriented and published in closed-access journals that are partly financed by advertising ^57^.

**E) Open access mandates and policies –** There are strong OA mandates, requiring scholars to make their outputs OA if based on publicly-funded research by following either the Gold or the Green OA route. Scholars are granted embargo periods of 6 or 12 months to comply with the latter ^13^. Besides public funders, CERN and the Sponsoring Consortium for OA Publishing in Particle Physics (SCOAP) play leading roles in promoting OA. SCOAP is an international partnership that aims to provide funding for the conversion of high-energy physics journals to OA. Within this scheme, libraries and research centers either pay reduced subscription fees for participating journals or stop paying altogether. Saved monies are used to pay publishers up front to publish OA articles ^60^. This enables scholars to publish OA without straining own research funds ^61^. CERN requires its scholars to publish their articles in journals covered by SCOAP. When circumstances require publication in other journals, APCs must be covered by funds from outside the CERN Budget. Where this is not possible, authors may request special permission and funds from CERN ^62^.

### Open access in the social sciences

The OA uptake in the social sciences is higher than in most disciplines of the humanities, but remains below the medical and natural sciences. For social scientists, open repositories appear to be of central importance for making research outputs OA. Gold OA, Hybrid OA and Bronze OA play a less important role ^1, 9, 11, 17, 18^.

**A) Author behaviour and attitudes –** Author surveys reveal that the awareness of OA publishing is low, and that OA publication outlets have not yet fully become part of the workflow for social scientists ^52, 63^. The knowledge of OA journals and repositories however appears to grow. Particularly young researchers report high levels of OA engagement ^63^. Most social scientists support the idea of OA in principle, but stringent quality control, improvement of the manuscript before publication and journal prestige appear to outweigh OA as journal selection criteria ^64, 65^. This adds to the fact that scholars and learned societies are concerned about the quality of peer review and editorial services in OA outlets ^66^. Of relevance is also that the monograph has a central place in the culture of publishing and is relevant to career advancement ^65, 67^. Monographs are less likely to be published OA because of authors’ concerns over restricted editorial services, difficulties in financing Book Processing Charges (BPCs) and doubts if unestablished OA publishers are able to translate authors’ efforts into reputational gain ^68^.

**B) Publisher behaviour and policies –** Few publishers have converted existing subscription journals to OA or set up new OA journals. Key journals remain closed. Amongst other factors, this relates to publishers fearing that authors will not be able fund APCs or that switching to OA will result in a loss of prestige ^65^. One notable exception is SAGE Open in 2011 – the OA mega journal model already popular in the natural and medical sciences ^69^. In addition, a few OA journals were launched by academic or professional societies ^51^. For some journals, such as the Historical Social Research, it has become common practice to make contents automatically OA after two years ^70^. In addition, a large variety of new economic models of OA publishing has emerged that offers viable alternatives to author-payment model. To name only two, this includes Knowledge Unlatched (KU) and the Open Library of Humanities (OLH) ^71^. Another innovative business model of OA publishing that has gained some popularity is the so-called “freemium” model, which makes HTML versions of articles and books openly available, while PDF and ePub formats are accessible only to subscribers ^72, 73^.

**C) Infrastructures of scholarly communication –** Some attempts have been made to promote repositories. Authors are now able to choose from more than 200 different OA repositories, the most of which are institutional or subject repositories ^74, 75^. Social scientists have however been slow to adopt Green OA, which might be because readers consider the article version of a manuscript as important and are likely to distrust versions of articles held in a repository ^76^. Institutional repositories predominantly host faculty working papers, while subject repositories have become part of the workflow for social scientists. Prominent examples are the Social Science Research Network, the Social Science Open Access Repository and the preprint server SocArXiv. Gold OA is also of little importance to social scientists. The few existing OA journals are restricted to highly specified sub-disciplines with limited impact and small readership ^77^.

**D) Structural and institutional factors –** Monographs are one of the main work products in the social sciences and highly relevant for academic career advancement. Besides author concerns over prestige and standards of editorial services of OA monograph publishers, the high costs and procedural complexities associated with producing monographs are important factors restricting the uptake on OA of monographs ^78^. In addition to this, social scientists have reported to face significant difficulties in access to grant funding for both APCs and BPCs, as most research in the social sciences is not done by means of project-specific funding ^32^.

**E) Open access mandates and policies –** Scholars in the social sciences face similar OA requirements as the natural and medical sciences, albeit with some special regulations. Monographs are generally not included in OA mandates. Most public funders only recommend OA for monographs. One of the few exceptions is the Swiss National Science Foundation (SNSF), which demands the OA publication of monographs and provides respective funding for BPCs ^68, 79^. The social sciences commonly also are granted longer embargo periods for archiving articles after publication in a subscription journal. While embargo periods of 6 or 12 months are the default for the natural and medical sciences, social scientists usually are granted 12 or 24 months ^13, 80^.

### Open access in the humanities

The OA uptake in the humanities is lower than in most other fields. For scholars, open repositories appear to be of greater importance than Gold OA journals ^9– 11, 17^. Not much information is available on the importance of Hybrid and Bronze OA. One recent study has indicated that Hybrid OA is of central importance for the humanities and that Bronze OA plays a similar role as Gold OA ^11^.

**A) Author behaviour and attitudes –** Authors operate within a symbolic economy of prestige that is usually among the prime motivations in choice of publication venue ^81^. The relative prestige of publications is determined by a scarcity correlation with a shortage of labour on hiring, tenure, and grant panels, although most humanities fields use an informal hierarchy of publications rather than quantitative measures such as the Impact Factor ^82^. Further, academics and learned societies have often been opposed to OA, for a variety of reasons that range from concerns to misunderstandings, worries about licensing and plagiarism, or fears for the standing of their members ^54, 83^. In addition to this, humanities scholars show fairly low levels of awareness of OA and OA publication outlets in their fields ^84^. That said, there are signs of a cultural shift with new economic models that do not rely on author payments, such as KU, OLH, Open Humanities Press, Open Book Publishers, Punctum Books, which appear to have some traction with some humanities scholars. It is tempting to posit that humanities scholars are less driven by technological change than counterparts in science disciplines, and thereby less inclined towards digital and open publishing solutions. A recent report however demonstrates that research and communication in the humanities are largely taking place in an electronic environment, which includes blogs or wikis, and that the distribution of scientific information occurs simultaneously through print and digital media, with the latter gaining importance ^85^.

**B) Publisher behaviour and policies –** The main concern driving humanities publishers is ongoing sustainability of their operations. In switching to an APC or BPC model, publishers fear that their academic authors will not be able to pay. It is also clear that highly selective publication models, which are common in the humanities, are more difficult to run, economically, on an OA basis. Hence there is little movement towards a fully Gold OA ecosystem, although it is unclear what impact the recently announced pan-European initiative, Plan S, may have upon this. That said, most humanities publishers are compliant with green OA mandates ^86^. On the other hand, some humanities scholars have argued that a longer citation half-life (particularly for monographs) should translate to longer embargo periods, although this does not necessarily match up to sales half-lives ^87^. Some publishers now offer Hybrid OA for their existing subscription journals ^81^. This allows authors to conform with most OA mandates while publishing their work in familiar journals by traditional publishers. This might explain why Hybrid OA is popular in these disciplines. Despite some disciplines having healthy cultures of offline working paper circulation (philosophy, for instance), preprints have not taken off. Policies on prior publication remain tight, especially in prestigious venues.

**C) Infrastructure of scholarly communication –** In addition to institutional repositories, there has been a growth of subject repositories, such as CORE, the Open Access Repository for the Humanities, which is operated by Modern Language Association of America. There has also been a prominent culture, for many years, of scholarled OA journal and book publications ^81^. There is no preprint infrastructure at a comparative scale to arXiv. Further, for long-form reading, print remains a crucial resource and scholars often report that they do not wish to read such works in a digital format.

**D) Structural and institutional factors –** The high costs of producing monographs are a key structural factor that limits OA ^67, 78^. Further, most research work in the humanities does not receive project-specific funding, making it difficult to integrate APCs into grants. That the humanities are often of lesser importance in institutional hierarchies also means that it can be difficult to secure funding. The slow cycle of producing long-form outputs is also problematic for OA, as the time investment (and hoped-for credit) is greater than those of a journal article, leading scholars into conservative behaviours. There are also substantial challenges around third-party rights and reuse of images, particularly within disciplines such as Art History, where it can be difficult to negotiate re-use rights for dissemination. Some disciplines, such as creative writing, have outward facing cultures that rely on sales, which works poorly under OA. The production of such outputs may have a research process behind them and various institutional policies will regard those as scholarly undertakings. The extent to which such work should be exempted from OA mandates remains an ongoing debate.

**E) Open access mandates and policies –** In national cultures, such as in the UK, the humanities face similar OA requirements as the social sciences, involving monographs being excluded from OA mandates and embargo periods of 12 or 24 months for the archiving of journal articles after publication in a subscription-journal. A few research foundations, such as the Wellcome Trust, will pay for Gold OA to monographs in the medical humanities. It appears likely, given recent moves among European funders, that policies around lengthened embargo periods for the humanities will be harmonized with other disciplines, e.g. Plan S, which does not allow any embargoes ^88^.

### Open access in law

The transition to OA of legal literature is in its infancy. Legal studies feature some of the lowest OA rates ^24^.

**A) Author behaviour and attitudes –** Scholars have been reluctant to adopt OA despite agreeing that the field would benefit from journals that publish OA ^89– 91^. Many authors either are not aware of OA or have little incentive to publish OA ^92^, but the field is slowly moving with networks for OA being established, such as the German-speaking network jurOA (established in 2018). It is common practice that academics and practicing lawyers publish in the same legal journals or commentaries. Some practicing lawyers might even prefer to publish in law journals behind paywalls, thereby guaranteeing exclusive access to their knowledge ^93^. Because of the high relevance of national legal systems, large parts of the literature are written in the languages of these countries and published in journals or books operated in the same countries. The argument that OA enables worldwide readership is of limited relevance. On the other hand, many legal issues are of interest not only to legal scholars but also to the media and politics ^94^. The role of electronic media in supporting scholarly communication and dissemination of research findings is growing but important databases (e.g. HeinOnline in the United States or BeckOnline in Germany) are paywalled ^95^.

**B) Publisher behaviour and policies –** In the U.S., many law reviews are published by law schools, not by for-profit publishers ^95– 97^. In contrast to commercial publishers, law schools do not have the usual incentives to oppose OA and a growing number of their journals are converted to OA. This is different in jurisdictions outside the US where legal scholarship is generally published by commercial publishers ^89, 97^. Due to the small demand for OA by legal scholars, there are little to no incentives for for-profit publishers to set up new OA journals or book series or to convert existing subscription journals to OA. There are some notable exceptions. In recent years, some OA law journals have been set up that are predominantly community-driven (e.g. Journal of Intellectual Property, Information Technology and Electronic Commerce Law and Forum Historiae Iuris in Germany or sui generis in Switzerland). According to the DOAJ, there are about 260 OA law journals. OA law journals from the US are in large part not listed, although it is not clear why this is the case. The Creative Commons List of OA Law Adopting Journals lists 37 OA law journals but most of the 17 Harvard Law School OA journals are missing ^98, 99^.

**C) Infrastructure of scholarly communication –** Most OA journals and open repositories are operated by universities and their law departments. Most universities in the U.S. have their own repositories and also publish their own legal OA working paper series. This idea gains some traction in other countries, for example in Germany, the Netherlands or Italy. Prominent examples of university-led OA journals involve Stanford Technology Law Review, Harvard Human Rights Journal, Bucerius Law Journal or the International Journal of Communications Law & Policy. There is only a limited number of disciplinary repositories and the uptake is slow. In the U.S. and in international law, the most popular disciplinary repository is SSRN, which is now owned by Elsevier. In English-speaking legal scholarship, scholars find it difficult to build reputation without being represented in SSRN ^100^. A growing number of universities is further providing support for setting up OA journals or transforming closed to OA journals (for example, by providing an Open Journal Systems infrastructure). Since practicing lawyers and scholars work almost exclusively with texts, OA infrastructures do not have to meet demanding technical requirements.

**D) Structural and institutional factors –** There are three types of work products: monographs, journal articles and commentaries covering a specific law. PhD theses are predominantly published as monographs and many universities routinely make PhD theses OA. While the authors of legal books are mostly academics, this is different for journal articles and legal commentaries where both academics and practitioners contribute. As a result, not only scholars and universities, but also practicing lawyers need to be convinced to move to OA. One possible way to foster OA might be to encourage academics and practitioners to publish in different journals and commentaries. Here, academics could publish in scientific OA journals and practitioners could keep using closed access journals and commentaries, which would be more practice-oriented.

**E) Open access mandates and policies –** OA mandates by public funding agencies and research foundations only have limited impact since legal research is relatively inexpensive and does not depend on third party funding in large parts ^96^. As law is often considered as a discipline related to the humanities, scholars in this field face the same OA requirements as the social sciences and humanities, including relatively long embargo periods for Green OA and monographs that are excluded from OA requirements. It can be assumed that OA mandates by universities will have a greater potential to foster change ^7^ An important alternative to top-down OA mandates are OA policies from law schools and non-binding statements promoting OA. In 2009, the directors of the law libraries of 12 US Universities signed the Durham Statement on OA to Legal Scholarship, which urges law schools to make their scholarship immediately available upon publication in stable, open and digital formats ^102^.

## Discussion

Many of the discussions surrounding OA revolve around how it affects publishing practices across academic disciplines. In the first part of this study, we reviewed eleven bibliometric studies that assessed OA publishing across broad academic disciplines and thereby identified discipline-specific OA publishing patterns. In the second part of this study, we explained these findings by examining a variety of data sources.

Over the last three decades, scholarly publishing has experienced a fundamental shift from closed access to OA. While there is little doubt over the notion that the proportion of scholarly literature that is openly accessible has increased continuously across all disciplines, the studies included in our review show great variation in terms of how much of the literature is OA. Estimated OA levels for publication years after 2010 varied between 29.4% and 66% ^13, 25^, with most studies reporting OA levels to lie somewhere between 50% and 60% ^10, 14, 17, 18^. In part, this variation could be explained by the fact that studies, which reported high OA levels, included ASNs and FA in their estimations. This caused OA shares to be overreported ^10, 17, 18^. Because most of these studies included ASNs and FA not as separate OA sub-type but as parts of "Green OA" or "Other free availability" together with other Hybrid and Bronze OA, it was not possible to quantify the size of overreporting. At the same time, Piwowar *et al.* (2018) and Bosman and Kramer (2018), who reported particularly low OA levels, used the oaDOI service to search for freely available full-text papers, which has been shown to be more conservative than methods used by other studies in our review, e.g. Archambault (2014). Their results should therefore be interpreted as minimum proportions of papers that are OA ^11, 25^.

The driving forces behind the transition of scholarly publishing towards OA are manifold and intertwined: First, bottom-up advocacy initiatives from within the scientific community promote the free access to scholarly outputs. Second, funding organisations, governments and universities implement strong OA mandates that require scholars across disciplines to make their research outputs OA. Third, at least for the medical, life and natural sciences, OA mandates are usually combined with convenient open repositories for depositing articles and with sufficient funds for covering fees for publication in OA journals. This finding is in line with other reviews that have identified the interplay between bottom-up and topdown factors as the driving force for OA ^6, 103^. Some reviews report the interplay between ’soft factors’, such as different degrees of awareness and cultures, and ’hard factors’, such as institutional barriers, as the main determinant of disciplinary OA publishing patterns ^104^.

Globally, most OA is published as journal articles in subscription journals for which the accepted or the published version can be retrieved from an open repository (Green OA). Publication of articles in pure OA journals (Gold OA) is also of importance, even though the relative uptake remains well below Green OA for most publication years ^1, 10, 11^. Evidence on the importance of the remaining OA routes is sparse as only three studies have determined respective uptake levels ^10, 11, 18^. Publication of articles on the journal or publisher website that are free to read without a clearly identifiable license (Bronze OA) is of similar importance as Gold OA. Publication of articles free to read in subscription based journals either under open licenses (Hybrid OA) or after embargo periods (Delayed OA) are of less relevance for OA publishing than Green, Gold and Bronze OA. ASNs and FA also are of importance for making research outputs openly accessible ^18^. Some of these routes are more open and sustainable than others. In general, the more a publication outlet allows for immediate readability and reuse and the more it guarantees long-term access to its contents, the more open and sustainable it is ^19^. Following this, Bronze OA, ASNs and FA are less open and sustainable than Green and Gold OA: As Bronze articles are not accompanied by a license, articles are free to read, but usually cannot be downloaded, redistributed or reused. Also, publishers may decide to change contents or to remove them entirely ^20^. Contents hosted on ASNs and personal websites are vulnerable to take-down notices by publishers due to potential copyright infringements. This is of concern as these sub-types feature high uptake levels.

The studies included in our review suffer from limitations in determining uptake levels for OA routes. First, some studies merged different OA sub-types, for example ASNs with Green OA ^1^, Gold with Hybrid and Bronze OA ^17^ or ASNs with Hybrid and Delayed OA ^10^. Also, studies did not assess Platinum OA as a separate OA route, but likely as part of Gold OA. As a result, estimates for some OA sub-types are overreported, which limits the comparability of studies. Second, for most studies, Green OA uptake levels are underreported ^1, 9, 11, 13, 14, 17, 18, 24, 25^. This is because databases like Scopus and WoS employ strict demarcations for Green OA as OA in the form of author submitted versions are not included. Also, not all repositories are harvested by these databases, so that Green OA contents are incomplete ^25^.

The shift of scholarly publishing towards OA occurs uneven across disciplines in two respects. First, scholars in different disciplines differ in how much they embrace OA. This manifests itself in varying proportions of openly accessible research outputs across disciplines. The medical sciences feature the highest levels of OA, closely followed by physics, mathematics, information technology and astronomy. OA uptake in the social sciences is below the medical and natural sciences, but remains above OA prevalence that we observed for the humanities and law. Chemistry and engineering feature OA levels comparable the humanities and law. Second, academic disciplines differ regarding the relative importance of publication channels used by scholars to publish OA. The Gold OA route is of central importance for the medical sciences, followed by Hybrid, Bronze, and, with some distance, Green OA. Green OA plays an important role for scholars in physics, mathematics, information technology and astronomy, while scholars in engineering and chemistry publish most OA through the Gold OA route. For social scientists, open repositories are of central importance, closely followed by publication in Gold OA journals, and, with some distance, Hybrid and Bronze OA. Most OA within the humanities is published as Hybrid OA, followed by Green OA, Bronze OA and Gold OA. A number of other studies agree with the notion that disciplines differ in their OA publishing behaviour: For example, Tomaszewski *et al.* (2013) showed that in the fields of sciences and medicine, the OA movement has been going on earlier than in humanities and social sciences ^105^. Similarly, Liu and Li (2018) found that both the social and natural sciences experienced OA growth, but note that the social sciences now feature a lower absolute quantity and relative share of OA publications ^104^.

Our study has several limitations. In our systematic review, the strictness of inclusion criteria caused studies to be left out that also analysed disciplinary OA publishing practices, albeit focusing on only one type of OA mechanism or one discipline. Further, most bibliometric studies included in our review assessed publishing practices across broad academic disciplines, which produced coarse-grained data. Differences in the OA uptake between sub-disciplines remain undetected. We encourage future research to take into account sub-disciplines. Further, the included bibliometric studies differed substantially in terms of their definitions of OA, included OA subtypes, covered publication years, employed search strategies for OA full texts and time-lags between when levels of OA was measured and when studied materials were published. We tried to account for this heterogeneity in our review. As for our narrative review, there is a chance that evidence has been selectively chosen. We tried to keep this to a minimum by using an analytical framework. Furthermore, we included author surveys to explain publishing behaviour. There might be discrepancies between what scholars self-report about their publishing preferences and what really drives their behaviour. Despite these limitations, our review is the first to comprehensively explain OA publishing patterns across academic disciplines. We identified patterns and trends of discipline-specific OA publishing practices and revealed barriers and potentials for OA across disciplines.

## Data availability

All data underlying the results are available as part of the article and no additional source data are required.

## Notes

^1^For an in-depth review of the literature on OA, see for example Tennant *et al.* (2017) ^6^.

^2^Of note, for analytical purposes of this article, ASNs and FA will be included in the results section.

^3^Figures for the humanities and the arts were not included due to underrepresentation of these disciplines in terms of WoS and DOI coverage

^4^The sum of shares for individual OA routes does not match with the overall OA figure because as we do not include FA in this table.

^5^The sum of shares for individual OA routes does not match with the overall OA figure because as we do not include FA in this table.

^6^For a comprehensive discussion of the merits of these perspectives in explaining publishing practices see Kling & Kim (2000) and Oostveen (2004).

^7^See for example swissuniversity guidelines addressed at Swiss higher education institutions for drafting own OA policies ^101^.
