# Supplementary material for: Discipline-specific open access publishing practices and barriers to change: an evidence-based review
Source: F1000Res. 2020 Mar 26;7:1925. Originally published 2018 Dec 11. [Version 2] doi: 10.12688/f1000research.17328.2 (PMC7194335; doi:10.12688/f1000research.17328.2)
Supplement: Peer review history

# Peer review: Reviewer response for version 2

**DOI:** 10.5256/f1000research.25075.r61709

**Published:** 2020-04-24

**Authors:** Birgit Schmidt

**Affiliations:** 1 Göttingen State and University Library, Göttingen, Germany

The revised version addresses all my comments very well and reads much clearer now. Therefore I am glad to approve the revised version.

 You may consider correcting reference #37 (as it currently links to a bookmark which in turn refers to a blog post), please use:

 Johnson, Rob, Fosci, Mattia, Chiarelli, Andrea, Pinfield, Stephen, & Jubb, Michael. (2017, February 28). Towards a Competitive and Sustainable OA Market in Europe - A Study of the Open Access Market and Policy Environment. Zenodo ^1^.

Is the work clearly and accurately presented and does it cite the current literature?

Partly

If applicable, is the statistical analysis and its interpretation appropriate?

Partly

Are all the source data underlying the results available to ensure full reproducibility?

Partly

Is the study design appropriate and is the work technically sound?

Yes

Are the conclusions drawn adequately supported by the results?

Partly

Are sufficient details of methods and analysis provided to allow replication by others?

Partly

Reviewer Expertise:

NA

I confirm that I have read this submission and believe that I have an appropriate level of expertise to confirm that it is of an acceptable scientific standard.

## References

[1] : Towards a Competitive and Sustainable OA Market in Europe - A Study of the Open Access Market and Policy Environment.2017; 10.5281/zenodo.401029 10.5281/zenodo.401029 Reference source

# Peer review: Reviewer response for version 2

**DOI:** 10.5256/f1000research.25075.r61711

**Published:** 2020-04-16

**Authors:** Mikael Laakso

**Affiliations:** 1 Hanken School of Economics, Helsinki, Finland

The authors have successfully improved the manuscript in version 2. The main questions and concerns I raised in my earlier review have been acknowledged and I can now give the manuscript the label of Approved.

Is the work clearly and accurately presented and does it cite the current literature?

Partly

If applicable, is the statistical analysis and its interpretation appropriate?

Not applicable

Are all the source data underlying the results available to ensure full reproducibility?

Yes

Is the study design appropriate and is the work technically sound?

Partly

Are the conclusions drawn adequately supported by the results?

Yes

Are sufficient details of methods and analysis provided to allow replication by others?

Yes

Reviewer Expertise:

scholarly publishing, open access, information systems science

I confirm that I have read this submission and believe that I have an appropriate level of expertise to confirm that it is of an acceptable scientific standard.

# Peer review: Reviewer response for version 1

**DOI:** 10.5256/f1000research.18948.r43518

**Published:** 2019-02-19

**Authors:** Birgit Schmidt

**Affiliations:** 1 Göttingen State and University Library, Göttingen, Germany

The article reviews and synthesises quantitative and qualitative findings on disciplinary OA publication practices and uptake over time. The article extensively reports figures on OA levels from former studies (selected based on a set of criteria) and discusses factors influencing disciplinary publishing cultures based on a framework of analytical dimensions. 

 Overall, this creates a useful comprehensive overview and reference point. 

**Is the work clearly and accurately presented and does it cite the current literature?**

 A few references should be reconsidered, e.g. on p. 11 the authors cite Kling and Kim (2000) for a statement that “social and cultural factors are believed to be of less or no importance in explaining the emergence of OA”. This does not seem justified as Kling and Kim’s study adopts a social shaping perspective, in order to analyse field differences in the use of electronic media.

 Some references are certainly not the most authoritative, e.g. websites that summarise disciplinary attitudes (e.g. reference 52). For further comments on references see the list below.

**Is the study design appropriate and is the work technically sound?**

 The authors search and selection criteria for studies on the uptake of OA are sound. However, in some cases that leads to limitations as several studies, in particular the earlier ones, only provide little information about the status for certain disciplines, e.g. the humanities. In such cases it would have been good to further amend the data by taking into account disciplinary studies. The authors note that there is little consistency in the reported uptake of OA, however, this should be discussed earlier and in more detail in the study.

**Are sufficient details of methods and analysis provided to allow replication by others?**

 The description of methods on how studies were retrieved and selected as well as the framework of the analysis seem sufficient. However, too little attention is given to major differences of the considered studies, in particular the definitions of OA used, the data sources (including how was OA identified), and how the limitations of the studies should be considered in the comparison of study results.

 The authors’ definition of the different OA routes is certainly not homogeneously applied by all selected studies, as they mainly follow Piwowar et al.’s (2018) ^1^ approach to define OA as exclusive categories. More commonly, “green OA” is defined as “self-archiving” of peer-reviewed works in OA repositories, i.e. green OA overlaps with other OA routes.

 Another deviation is e.g. the study by Archambault et al.(2014) (reference 10) which considers green OA as “OA provided before or immediately after publication by author self-archiving” while focusing on deposit in “institutional repositories and some thematic repositories listed in OpenDOAR and ROAR” (Ibid., pp. 4 and i), e.g. arXiv.org was included; PubMed Central was considered under Other OA. In particular, it can thus be expected that in this case preprints have been included.

 There are also a number of results in some of these studies which deserve further attention – and if possible explanation – in particular, if these have not been observed in other studies: e.g. the high rate of hybrid OA for Mathematics and the Humanities reported by Piwowar et al.(2018) ^1^. Another example are the surprisingly high gold OA figures found by Jamali and Nabavi (2015) ^2^, please make explicit how gold OA was defined.    

**If applicable, is the statistical analysis and its interpretation appropriate?**

 There is no statistical analysis across the selected studies, the authors mainly provide an overview of the reported levels of OA, and organise these in three phases. In general, the authors report all available figures of OA uptake by discipline but do not provide any comments on the sometimes vast differences of these figures.     

**Are all the source data underlying the results available to ensure full reproducibility?**

 The authors have created summary tables of relevant studies and their framework for the further analysis. Regarding the uptake of OA the authors refer to data in the selected studies but have not created additional comparative data. All additional information which was used in the narrative review of mechanisms and factors shaping OA publishing is disclosed through the list of references.

 Some of the considered studies are not reproducible based on the chosen methodology (e.g. often a web search for an openly available version of a research article was applied, which may change over time). 

**Are the conclusions drawn adequately supported by the results?**

 Yes, overall the main conclusions are adequately supported.

 There are in addition a couple of other issues and small errors which I would like to point out:

- Typos: “66& for publication years…” - use %; replace “SEM” by “STM” for “Science, Technology and Medicine”; replace “PLoS” by “PLOS”.
- p. 4: The conclusion regarding the comparison of OA levels for medical research areas and the humanities is not quite convincing as only one figure for the humanities (based on Gargouri et al., 2012 (see reference 9)) is provided.
- Table 2: Consider adding more information on the definition of OA or a specific column which explains how the study deviates from the definition of OA provided by the authors. It is also important to note which studies treat the definitions as exclusive categories and which used definitions of OA that allow overlaps, in particular with green OA. Add “with DOIs” in column two of Piwowar et al (2018).
- The reference European Commission (2014) should be cited as Archambault et al. (2014).
- Table 3: The second column on Piwowar et al. (2018) should include a note that the figures for the Humanities and the Arts were not included as these disciplines are underrepresented in the WoS and in terms of DOI coverage. In the Hybrid OA column the overall figure cannot be correct: 0.6% is too low; according to Table 3 of Piwowar et al. it should be replaced by 4.3%. Even if certain routes of OA have not been assessed, e.g. Hybrid and Bronze OA, it would be good to add a note if they have been included in other categories. Please check the figures provided for Martín-Martín et al. (2018) ^4^, the overall OA figures do not seem to match with the sum of the OA routes (the sum is substantially smaller, what is missing? E.g. Social and Behavioral Sciences: 49.9% OA vs. a sum of 23.4% for all OA routes). For further studies the overall OA figures do not match with the sum of the OA routes either, e.g. for Science Metrix (2018) ^4^ and Jamali and Navabi (2015) ^5^, please explain.
- p. 10: Please correct the underestimation of Hybrid OA, as noted above the overall figure found by Piwowar et al.(2018) ^1^ is 4.3% for recent articles with a DOI in WoS. 
- p. 11: The statement “… as the OA model is unlikely to generate the level of income and profit that can be achieved with the subscription model” deserves a reference.
- p. 11: “This also applies to academic and professional societies” – the given reference does not seem to be connected to this statement.
- Table 4: “APC levels” is a bit narrow as a perspective, and not much is said about cost aspects in the later discussion. Regarding infrastructures the support aspect is missing (e.g. institutional support for the green and gold OA routes). Regarding structural and institutional factors copyright is missing (this is not just an aspect which is relevant for publishers).  
- p. 12: NIH OA mandate – mention the year in which the mandate was introduced
- p. 13 E) does not mention Wellcome Open Research
- p. 13 A) Preprints do have a tradition in biology, but were mainly circulated in small circles of colleagues. Please note that on arXiv.org quantitative biology represents a very small share and is not representative for the discipline. A reference for molecular biology is e.g. the study of Kling and Kim (2000) ^6^. Thus the conclusions on green OA do not fully apply to biology.   
- p. 14 C) You provide several examples of OA journals in Chemistry, what does “enjoy popularity” mean here?
- p. 14 D) Please provide a reference for the stated national focus of engineering.
- p. 14 E) Remove bold face for the word “Reflecting”.
- p. 14: There seems to be some redundancy in the introduction to “Open access in the social sciences”. Moreover, please add a reference for the order of OA routes as the one you state is not found by the most recent studies (compare e.g. Piwowar et al ^1^.). 
- p. 14 A) Reference 51 seems a bit old for a statement about the present state.
- p. 15 – typo “Open library of Humanities” should be “… Library …”
- p. 15: “While long-term access to research outputs is questionable in these models…” – this is an unjustified statement.
- p. 15 C) You do not provide any evidence for *growth* of the number of repositories when you state the current status, please justify this statement.
- p. 15 C) “Because OA preprint repositories do not employ peer review, however, social scientists have been slow to adopt Green OA.” – This statement is not convincing, preprint servers circulate non-peer reviewed versions while green OA focuses on final author manuscripts (after peer review) or the publisher’s version (i.e. you compare apples and pears here).
- p. 15 C) Some of the details about academic journals could be moved to B).
- p. 16 OA in the humanities: Add reference to the cited order of green OA, Bronze OA etc.
- p. 16 A) The last sentence should be amended, otherwise it is just a cliché pointed out but not put to rest.
- p. 16 C) MLA Commons is a network of scholars, the related repository is called CORE.
- p. 16 C) You state that “post-publication peer review remains rare … with a few notable notable exceptions and experiments”. – Please note that one of your references do not support this statement: Bourke-Waite (reference 83) does not elaborate on post-publication peer review but points out that for the HSS double-blind peer review remains the norm and notes an experiments of open peer for monographs by Palgrave-Macmillan. Brienza (2012) ^7^ certainly fits here as for the book of K. Fitzpatrick post-publication peer review was applied to a draft version (in the sense of open peer commenting). It could be argued that review articles which are quite common for monographs to be considered as a form of post-publication peer review.
- p. 16 E) For the last sentence you may add an indicative reference, e.g. to Plan S which does not allow any embargoes.
- p. 17 OA in law: Please add references to the statement that legal studies feature some of the lowest OA levels.
- p. 17 B) JIPITEC is based in Germany (which is in the EU…). You note that several journals are not listed in the DOAJ which is indeed a pity, they may not have re-registered after the introduction of revised criteria in 2014.
- p. 18 “overall OA levels well above 50%” – add reference, not all recent studies agree on this point; “the relative uptake on Gold OA remains well below Green OA for most publication years and disciplines” – add reference; “hybrid OA generally is of little variance” – not according to Piwowar et al. (2018); “hybrid OA… with 1% or less of all scholarly outputs” – this figure is too low, Piwowar et al. (2018) found 4.3% hybrid OA on average
- p. 18 “… biology were the early pioneers” – see comment on biology above. OA in biology is not initially strong via preprints and green OA (arXiv.org only holds a limited number of quantitative biology papers), uptake gets stronger via gold OA with the wave of new OA journals in the 2000s, and only from 2013 onwards preprints finally take off (launch of PeerJ Preprints and bioRxiv). For a discussion of earlier initiatives/experiments with biology preprints which were pretty much blocked by the publishing industry see: Cobb, M. (2017). The prehistory of biology preprints: A forgotten experiment from the 1960s ^8^.
- p. 18 “Barriers to OA in chemistry and engineering can be identified as concerns about the quality of OA journals” – add reference
- p. 19 “difficulties in assessing funding for APCs and BPCs” – you mean “accessing” here, please also add a reference.
- p. 19 “we observed signs of cultural change particular in young scholars” – add reference
- p. 19 “Most OA within the humanities is published as Hybrid OA…” – try to explain why hybrid OA comes first. Piwowar et al. (2018) which seems to be the source here do not seem to do.
- p. 19 “This includes models such as OLH or other crowd funding initiatives, such as KU.” – You may consider adding a reference here, e.g. Bulock, C. (2018) ^9^.
- p. 19 “… OA … is shaped by the scholars that use respective communication technologies.” – You do not really discuss different types of communication technologies but mainly point out common communication formats, channels and infrastructures.

Is the work clearly and accurately presented and does it cite the current literature?

Partly

If applicable, is the statistical analysis and its interpretation appropriate?

Partly

Are all the source data underlying the results available to ensure full reproducibility?

Partly

Is the study design appropriate and is the work technically sound?

Yes

Are the conclusions drawn adequately supported by the results?

Partly

Are sufficient details of methods and analysis provided to allow replication by others?

Partly

Reviewer Expertise:

library and information science, scholarly communication, open science

I confirm that I have read this submission and believe that I have an appropriate level of expertise to confirm that it is of an acceptable scientific standard, however I have significant reservations, as outlined above.

## References

[1] : The state of OA: a large-scale analysis of the prevalence and impact of Open Access articles. *PeerJ*.2018;6: 10.7717/peerj.4375 e4375 10.7717/peerj.4375 29456894PMC5815332

[2] : Open access and sources of full-text articles in Google Scholar in different subject fields. *Scientometrics*.2015;105(3) : 10.1007/s11192-015-1642-2 1635-1651 10.1007/s11192-015-1642-2

[3] : Evidence of Open Access of scientific publications in Google Scholar: a large-scale analysis.2018; 10.31235/osf.io/k54uv 10.31235/osf.io/k54uv

[4] : Open access availability of scientific publications.2018;1-36 Reference source

[5] : Exploring human resource management roles in corporate social responsibility: the CSR-HRM co-creation model. *Business Ethics: A European Review*.2015;24(2) : 10.1111/beer.12085 125-143 10.1111/beer.12085

[6] : Not just a matter of time: Field differences and the shaping of electronic media in supporting scientific communication. *Journal of the American Society for Information Science*.2000;51(14) : 10.1002/1097-4571(2000)9999:9999<::AID-ASI1047>3.0.CO;2-T 1306-1320 10.1002/1097-4571(2000)9999:9999<::AID-ASI1047>3.0.CO;2-T

[7] : Kathleen Fitzpatrick: Planned Obsolescence: Publishing, Technology, and the Future of the Academy. *Publishing Research Quarterly*.2012;28(2) : 10.1007/s12109-012-9271-6 150-151 10.1007/s12109-012-9271-6

[8] : The prehistory of biology preprints: A forgotten experiment from the 1960s. *PLoS Biol*.2017;15(11) : 10.1371/journal.pbio.2003995 e2003995 10.1371/journal.pbio.2003995 29145518PMC5690419

[9] : Crowdfunding for Open Access. *Serials Review*.2018;44(2) : 10.1080/00987913.2018.1472477 138-141 10.1080/00987913.2018.1472477

# Response

**Published:** 2020-03-26

**Authors:** Anna Severin

**Affiliations:** Swiss National Science Foundation, Switzerland

We would like to thank Birgit Schmidt for her useful peer review of our study. In response to her comments, we have made the following revisions throughout the manuscript:

- We agree that the reference Kling and Kim (2000) on p.11 should have been reconsidered. Shortening and restructuring of the paper removed the statement from the text altogether. As for references that were not authoritative (e.g. reference 52), we either replaced them entirely or included additional references.
- We agree that we might have excluded important studies on the status of OA for individual disciplines. We now take into account disciplinary studies in the narrative review and in the Discussion section, thereby framing the results of the meta-synthesis. We now discuss the heterogeneity of included studies and how this might affect the consistency and comparability of study results (please see Methods, Results, Discussion).
- We now discuss the heterogeneity of included studies and how this might affect the comparability of study results (please see Methods, Results, Discussion).
- We have added a sub-section in which we state our definition of OA and its routes. In Table 2, we have added a column on the OA definition used by each study included in our review. Throughout the Results and the Discussion section, we note if included bibliometric studies differ from our definition of OA. In the Results section, we highlight how studies differed from one another in their definitions of OA and its sub-types. In the Discussion, we highlight the consequences thereof for the comparability of study results. In the narrative review, we now offer potential explanations for the popularity of Hybrid OA in specific disciplines.
- We have corrected the following typos: “66& for publication years…” - use %; replace “SEM” by “STM” for “Science, Technology and Medicine”; replace “PLoS” by “PLOS"
- Due to sparse data on the early uptake on OA in the humanities, we have removed the conclusion regarding the comparison of OA levels for medical research areas and the humanities (p. 4 in version 1). We note this limitation in the narrative review.
- In Table 2, we have added a column on the OA definition used by each study included in our review. Throughout the Results and the Discussion section, we note if included bibliometric studies differ from our definition of OA.
- We added "with DOIs" in column two of^Piwowar et al (2018).
- We have cited the reference European Commission (2014)  as Archambault et al. (2014).
- Table 3 (now Table 4): 
  - In a footnote, we added the information that for Piwowar et al. (2018), the figures for the Humanities and the Arts were not included as these disciplines are underrepresented in the WoS and in terms of DOI coverage.
  - We have corrected the Hybrid OA overall figure for Piwowar et al. (2018).
  - We have corrected the Hybrid OA overall figure for Piwowar et al. (2018), as well as the figures for Jamali and Navabi (2015).
  - As for Martin-Martin et al. (2018) and Archambault et al. (2014), the sum of shares for individual OA routes does not match with the overall OA figure because both studies included Free Availability / Other OA in their estimation of OA levels – which this study does not. For both studies, we have noted this in footmarks and are discussing it in the Results and Discussion section.
- We have added two references to the statement  “… as the OA model is unlikely to generate the level of income and profit that can be achieved with the subscription model”: Hagner (2008) and Johnson et al. (2017).
- For the statement “This also applies to academic and professional societies”, we have replaced the reference, now: Albert (2006).
- Table 4 (now Table 5): We have replaced “APC levels” with “publishing costs”. We also added more information on support aspects and copyright factors in Table 4.
- We now mention the year in which the NIH OA mandate was introduced.
- We now mention Wellcome Open Research.
- The remarks on OA and preprints in biology are now more differentiated.               
- We agree that we should have elaborated on what “enjoy popularity” means in the context of OA journals in Chemistry. Shortening and restructuring of the paper however removed the entire statement from the text.
- We now provide two references for the stated national focus of engineering: Rostan et al. (2014) and Kyvik & Ingvild (2017)S Kyvik and R Ingvild.
- We have removed the bold face for the word "Reflecting".
- We have removed the redundancies in the introduction o “Open access in the social sciences”. and added more references for the order of OA routes.
- We have added a more recent reference for a statement about the present state of open access in the humanities: Rodriguez (2014).
- We have corrected the typo “Open Library of the Humanities"
- We have removed the stament "While long-term access to research outputs is questionable in these models…”
- We have rephrased the statement on growth of repositories in the social sciences to   “Some attempts have been made to promote repositories in the social sciences” and provided two references for it: OpenDOAR database and Xia (2007). 
- We have rephrased the statement “Because OA preprint repositories do not employ peer review, however, social scientists have been slow to adopt Green OA.” and now provide a reference for it.
- We have added references to the cited order of OA routes for the humanities.
- We have corrected the statement on MLA Commons.
- Shortening and restructuring the paper removed the following statement from the text: “post-publication peer review remains rare … with a few notable notable exceptions and experiments”.
- We have added an indicative reference for the statement that Plan S does not allow any embargoes.
- We have added references to the cited order of OA routes for law.
- We have corrected the statement that JIPITEC is based in the EU.
- We have corrected the following statements: “overall OA levels well above 50%”, “hybrid OA generally is of little variance”, “hybrid OA… with 1% or less of all scholarly outputs”
- We have added more detailed information on preprint uptake in biology to the section on OA in biology and added a respective reference: Kaiser (2017).
- Shortening and restructuring of the paper removed the following statement from the text: “Barriers to OA in chemistry and engineering can be identified as concerns about the quality of OA journals”
- Shortening and restructuring of the paper removed the following statement from the text: "difficulties in assessing funding for APCs and BPCs”.
- Shortening and restructuring of the paper removed the following statement from the text: “we observed signs of cultural change particular in young scholars”
- Shortening and restructuring of the paper removed the following statement from the Discussion: “Most OA within the humanities is published as Hybrid OA…”. We however give a potential explanation for the popularity of Hybrid OA in the humanities in our narrative review.
- We added the reference Bulock, C. (2018) to the following statement: “This includes models such as OLH or other crowd funding initiatives, such as KU.”
- Shortening and restructuring of the paper removed the following statement from the text: "… OA … is shaped by the scholars that use respective communication technologies.”

# Peer review: Reviewer response for version 1

**DOI:** 10.5256/f1000research.18948.r43517

**Published:** 2019-02-12

**Authors:** Mikael Laakso

**Affiliations:** 1 Hanken School of Economics, Helsinki, Finland

The manuscript tackles a very important and ambitious topic, that of improving our knowledge about what differences there are in open access uptake across academic disciplines. A lot of bibliometric work has been done in this area, however, most of it has been fragmented as definitions and methodological approaches have varied a lot across studies. This study makes a welcome exception to most of the research within the field in not just producing yet another measurement of OA that is improved in some incremental way, yet failing to be compatible with results of earlier studies, but rather leverages what is already out there (both in terms of existing studies, but also other knowledge) in order to thoroughly discern how disciplines differ in their approaches to utilising various forms of OA. The manuscript has two main components 1) a systematic literature review of bibliometric research (which includes 11 articles), and 2) an analysis of open access in academic research disciplines interpreted through the theoretical lens of Social Shaping of Technology. I could easily see both parts being published as individual articles based on what they aim to achieve and in how challenging they are to put together, having them together like this is not a major problem but something that requires effort and rigour which this first version of the manuscript succeeds with to a satisfactory degree. The text itself is of high quality.

**Is the work clearly and accurately presented and does it cite the current literature?**

 For the most part, yes, but I do think the strictness of the criteria for the systemic literature review of OA uptake requires that supplementing research that is left out of the review is still discussed/reflected upon as in some other parts of the manuscript. I have a couple of recommendations for this that are mentioned below in this section.

 One source which I think is a great omission and gives a lot of detailed breakdown into the differences between OA journal differences is: Crawford, W. (2018). GOAJ3: Gold Open Access Journals 2012-2017. https://walt.lishost.org/2018/05/goaj3-gold-open-access-journals-2012-2017/. If “top-down” studies, focusing on only one type of OA mechanism, were excluded this study was perhaps not included on such grounds but I think it is doing the study a disservice – there is no better source that describes the disciplinary differences longitudinally across disciplines, including information about article processing charges, than that e-book and associated dataset. If not integrated into the meta-analysis it should at least be used in the other parts of the manuscript to frame the study and its results.

 Further reference you could consider, purely based on the idea that they have also explored disciplinary differences in the OA context specifically, albeit through analysis of bibliographic indexes:

 Liu, W. and Li, Y. (2018), Open access publications in sciences and social sciences: A comparative analysis. Learned Publishing, 31: 107-119. doi:10.1002/leap.1114 ^1^

 Ennas, G. and Diguardo, M.C. (2015), “Features of top-rated gold open access journals: an analysis of the Scopus database”, Journal of Informetrics, Vol. 9 No. 1, pp. 79-89 ^2^.

 Gadd, E., & Covey, D. T. (2019). What does ‘green’ open access mean? Tracking twelve years of changes to journal publisher self-archiving policies. *Journal of Librarianship and Information Science*, *51*(1), 106–122 ^3^

 In general I avoid suggesting citing material that I have been involved in authoring as part of reviews I have conducted, but in this case I would like to point out two studies that give precise metrics of various types of OA in narrowly defined disciplines, and another study which deals with disciplinary differences in self-archiving rights, utilizing these references is completely optional and not something that influences my verdict or recommendation for a revised version of the paper:

 Laakso, M. & Polonioli, A. Scientometrics (2018) 116: 291 ^4^.

 Laakso, M., & Lindman, J. (2016). Journal copyright restrictions and actual open access availability: A study of articles published in eight top information systems journals (2010–2014). Scientometrics, 109(2), 1167–1189 ^5^.

 Laakso, M. (2014), “Green open access policies of scholarly journal publishers: a study of what, when, and where self-archiving is allowed”, Scientometrics, Vol. 99 No. 2, pp. 475-492 ^6^.

 Table 3, being split onto 4 pages, is massive and very hard to use for making any conclusions between time/discipline(which each study having their own way of classifying as well)/OA method by eye. It is functional but far from optimal. In this case I would save a table like this to become an appendix, and rather compose a figure where the discipline categories have been standardized according to some well established scheme that fits well with most of the studies. This would come at the cost of precision in losing sub-discipline breakdowns in many cases but in my view that is worth the cost.

 I would also suggest to focus less on comparisons of decimal point-accuracy prevalence of OA mechanisms between the previous studies, since they vary so much depending on other factors than inherent disciplinary differences. Zooming out would make it easier to see, and tell the reader, what is important to focus on, not just drop the decimal points but also consider putting in subheadings or structuring the “Prevalence and patterns of open access publishing practices: Meta-synthesis of bibliometric studies” so that each “era” of OA development would get its own mini-narrative, now its just a long single block of text and a lot of percentages that are hard to relate to anything.

 The influence of academic social networks is in my view underrepresented in the review of existing literature and conclusions of the study, they have provided a substantial share of the OA copies measured in the various bibliometric studies and many authors also perceive them as essentially “solving” the issue of OA and paywalls on a personal level since there has been very weak monitoring of adherence to copyright on such services.

 The concept of Bronze OA would need further unpacking since in most of the reviewed studies it is present, but not always separated and referred to as such from other OA provision mechanisms.

**Is the study design appropriate and is the work technically sound?**

 The collection process for inclusion of existing literature contains both strict elements (specific indexes were queried with specific identical keywords, studies had to fulfil four pre-set criteria to be included) but also what seems like a liberal and flexible amount of bottom-up/explorative elements (authors contributing discipline specific OA findings/literature, scouring reference lists and Google Scholar profiles). This large jump between very strict and transparent, to a largely undocumented part where “anything goes” which has very little transparency other than the disciplinary analysis´ themselves could be expanded somewhat.

 For me it was a bit unclear what the first criteria in Table 1 when strictly applied entails, do the studies have to explore OA availability “bottom-up” through web-search engines/querying and giving uptake metrics for various OA mechanisms in one single study? If this is the case, which it could be by looking at the included studies, the criteria description should in my view be revised to communicate this.

 The time-lag between when a study has measured the level of OA and when the materials being measured were published, varies a lot across the included studies. I think this caveat/feature could be highlighted more in the text because it matters quite a lot if an article was searched for 1 year after it was published or 5 years after it was published.

**Are sufficient details of methods and analysis provided to allow replication by others?**

 Yes, the study is literature-based with no need for further data.

**If applicable, is the statistical analysis and its interpretation appropriate?**

 Not applicable

**Are all the source data underlying the results available to ensure full reproducibility?**

 Yes, the study is literature-based with no need for further data.

**Are the conclusions drawn adequately supported by the results?**

 Yes, my revision suggestions concern mainly minor points not critical to the main results and contribution of the study.

 The second half of the manuscript, which comprises the discipline-specific description of OA practices, I have very little to comment about since I think it does a great job at mixing research results with discipline-specific knowledge. The most central things are brought up and argued for well.

Is the work clearly and accurately presented and does it cite the current literature?

Partly

If applicable, is the statistical analysis and its interpretation appropriate?

Not applicable

Are all the source data underlying the results available to ensure full reproducibility?

Yes

Is the study design appropriate and is the work technically sound?

Partly

Are the conclusions drawn adequately supported by the results?

Yes

Are sufficient details of methods and analysis provided to allow replication by others?

Yes

Reviewer Expertise:

scholarly publishing, open access, information systems science

I confirm that I have read this submission and believe that I have an appropriate level of expertise to confirm that it is of an acceptable scientific standard, however I have significant reservations, as outlined above.

## References

[1] : Open access publications in sciences and social sciences: A comparative analysis. *Learned Publishing*.2018;31(2) : 10.1002/leap.1114 107-119 10.1002/leap.1114

[2] : Features of top-rated gold open access journals: An analysis of the scopus database. *Journal of Informetrics*.2015;9(1) : 10.1016/j.joi.2014.11.007 79-89 10.1016/j.joi.2014.11.007

[3] : What does ‘green’ open access mean? Tracking twelve years of changes to journal publisher self-archiving policies. *Journal of Librarianship and Information Science*.2019;51(1) : 10.1177/0961000616657406 106-122 10.1177/0961000616657406

[4] : Open access in ethics research: an analysis of open access availability and author self-archiving behaviour in light of journal copyright restrictions. *Scientometrics*.2018;116(1) : 10.1007/s11192-018-2751-5 291-317 10.1007/s11192-018-2751-5

[5] : Journal copyright restrictions and actual open access availability: a study of articles published in eight top information systems journals (2010–2014). *Scientometrics*.2016;109(2) : 10.1007/s11192-016-2078-z 1167-1189 10.1007/s11192-016-2078-z

[6] : Green open access policies of scholarly journal publishers: a study of what, when, and where self-archiving is allowed. *Scientometrics*.2014;99(2) : 10.1007/s11192-013-1205-3 475-494 10.1007/s11192-013-1205-3

# Response

**Published:** 2020-03-26

**Authors:** Anna Severin

**Affiliations:** Swiss National Science Foundation, Switzerland

We would like to thank Mikael Laakso for his helpful comments. In response to his review, we have made the following revisions throughout the manuscript:

- We agree that the strictness of inclusion criteria for the systematic review might have caused studies to be left out that also analysed disciplinary OA publishing practices, albeit from a different perspective. We acknowledge this limitation in the Discussion. To frame our results, we now include disciplinary studies in the narrative review and in the Discussion section. This includes some of the references you provided us with – thank you.
- We agree that Table 3 (now Table 4) is large and we would have liked to move it to the appendix too. Unfortunately, F1000 no longer supports supplementary files / appendices and requires all results to be kept as part of the main text. We therefore kept Table 3 (now Table 4) as is. To keep the paper short, we did not add any further figures or tables.
- We restructured and shortened the text significantly. We now focus less on comparisons of decimal point OA prevalence levels and only highlight the most significant figures. We further restructured the section “Prevalence and patterns of open access publishing practices: Meta-synthesis of bibliometric studies” and included more subheadings. We now illustrate the different phases / eras of OA development in a clearer structure, albeit without sub-headings for the different eras (as we believed this to be too fine-grained).
- We now discuss the importance of social networks and so-called “free availability” for making scholarly publications openly accessible (please see Methods, Results and Discussion section).
- We now define Bronze OA and discuss how it is defined and measured by the studies included in our review (please see Table 1, Table 3, Results and Discussion section).
- To address the fact that the collection process included both strict alements and a more liberal amount of explorative elements, we have added information on how we conducted the narrative review of discipline-specific OA literature (please see Methods). Further, we now state that the strictness of inclusion criteria for the systematic review might have caused studies to be left out that also analyzed disciplinary OA publishing practices. We also state that, as for our narrative review, there is a chance that evidence has been selectively chosen (please see Discussion)
- Yes, studies had to explore OA availability “bottom-up” through web-search engines/querying and giving uptake metrics for various OA mechanisms. We have added this information as a criterion for inclusion in our review (please see Table 1 and Methods).
- The discussion now includes the time-lag between when a study has measured the level of OA and when the materials being measured were published as a limitation to our study.

# Peer review: Reviewer response for version 1

**DOI:** 10.5256/f1000research.18948.r42144

**Published:** 2019-02-05

**Authors:** Richard Smith

**Affiliations:** 1 International Centre for Diarrhoeal Disease Research, Bangladesh (ICDDR,B), Dhaka, Bangladesh; 2 Patients Know Best, London, UK

I’m sorry that I haven’t been slow with reviewing this study, but the increasing length of studies, which generally I think necessary, makes peer review even more onerous.

**Importance:**

- The study asks two important questions - what is the variation in use of open access by discipline, and what might be the reasons for the differences? And it provides answers that are generally convincing, although hard to digest.

**Originality:**

- Individual studies provide some answers to these questions, but this review brings them together. That is useful.

**Methods and presentation:**

- Although the study is described as a meta-synthesis, it seems to me that it’s essentially a descriptive study. The authors find the studies and then largely describe what they report. There isn’t much synthesis, and I, as a reader, would appreciate more.
- There is huge heterogeneity in the studies of proportions of articles that are open access, but readers are left to look through a table that is four pages long - and largely to wonder for themselves about the reasons for the big variations. I know that all the data are there, but how is it that a study of 12m article from 2018 finds that 66% of articles are open access, whereas another equally large study from the same year finds that 29% are open access? Clearly they must be using different definitions of open access, and the authors don’t themselves define open access or its subsets (gold, green, hybrid, and bronze). They should. I’d like to see a summary table that has the following columns: Year of study, number of articles in the study, definition of open access, proportion of articles that were open access.
- There should be another summary table that shows the variation by discipline in overall open access rates and in the subsets of open access.
- Another useful addition would be a figure that showed how proportions of articles that are open access by discipline have varied over time.
- The authors might also give a table that shows the association between their “analytical dimension” and the different disciplines. At the moment readers can get this information only by ploughing through pages of text that describe the variations by discipline.
- As this is essentially a descriptive study I don’t have great criticisms of the methods and conclusions, although I recognise the weakness of surveys, which give us data on what people say about their behaviour than what really drives their behaviour.
- My biggest criticisms are about presentation. I’d like to see not only more tables and a figure but also a much shorter paper backed up by supplementary material if necessary. That would, I judge, make for a much more readable and useful paper and dramatically increase the number of readers.

**Minor points:**

- There is at the moment a great deal of repetition. The discussion is mostly a repetition of the results.
- I’d like to see some data in the abstract.
- I believe that abbreviations are a plague in medical writing, and I’d avoid them, including OA for open access.
- I’d drop “evidence-based” in the title.
- It would be good to have the search strategy as a table.
- Much of what’s in the results should ideally be in the discussion.
- There are a fair few typos.

Is the work clearly and accurately presented and does it cite the current literature?

Partly

If applicable, is the statistical analysis and its interpretation appropriate?

Not applicable

Are all the source data underlying the results available to ensure full reproducibility?

Yes

Is the study design appropriate and is the work technically sound?

Partly

Are the conclusions drawn adequately supported by the results?

Partly

Are sufficient details of methods and analysis provided to allow replication by others?

Partly

Reviewer Expertise:

Peer review, journalology, NCD, research misconduct. global health

I confirm that I have read this submission and believe that I have an appropriate level of expertise to confirm that it is of an acceptable scientific standard, however I have significant reservations, as outlined above.

# Response

**Published:** 2020-03-26

**Authors:** Anna Severin

**Affiliations:** Swiss National Science Foundation, Switzerland

We thank Richard Smith for his helpful review of our manuscript. In response to his comments, we have made revisions throughout version 2:

- Methods and presentation: In an attempt to restructure the paper, we have included more synthesis and less description of results. 
- We now discuss the heterogeneity of the studies included in our review (in terms of their methodological approaches and results) throughout the paper (please see Methods, Results and Discussion). We have added a sub-section in which we state our definition of OA and its routes (please see Methods and Table 1.) In Table 2, we have added a column on the OA definition of each study included in our review. Throughout the Results and the Discussion section, we note if included bibliometric studies differ from our definition of OA.
- Unfortunately, F1000 does not support supplementary files / appendices and requires all results to be included as part of the main text of the paper. This means that Table 3 (now Table 4) could not be moved to the supplementary files. In order to keep the paper short, we therefore decided not to add any further tables or images. Information on year of study, number of articles in the study, definition of open access is given in Table 2. Information on proportions of articles that were open access and in the subsets of open access is included in Table 3.
- Where appropriate, we discuss how the methodological heterogeneity of the studies included in our review affects the consistency and comparability of their results (please see Methods, Results and Discussion).
- We restructured and shortened the text significantly. We now illustrate the analytical dimensions (i.e. the different phases / eras of OA development) in a clear structure, albeit without a separate table.
- We now acknowledge the weakness of author surveys as a limitation (please see Discussion).
- We removed repetitions, including the repition of results in the discussion.
- We added data to the abstract.
- To keep the paper short, we decided to keep the abbreviations, including OA for open access.
- We dropped “evidence-based” in the title.
- We added the search strategy as a figure (please see Figure 1).  
- We moved parts of the results to the discussion and removed repetitions in both chapters.
- We corrected all typos.
